# Feasibility, acceptability and prognostic value of muscle mass and strength measurement in patients with hip fracture: a systematic review

**DOI:** 10.1007/s41999-024-01102-x

**Published:** 2024-11-29

**Authors:** James Prowse, Sharlene Jaiswal, Jack Gentle, Antony K. Sorial, Miles D. Witham

**Affiliations:** 1 AGE Research Group, Faculty of Medical Sciences, Translational and Clinical Research Institute, Newcastle University, Newcastle Upon Tyne, UK; 2 NIHR Newcastle Biomedical Research Centre, Newcastle Upon Tyne NHS Foundation Trust, Cumbria Northumberland Tyne and Wear NHS Foundation Trust and Newcastle University, Newcastle Upon Tyne, UK; 3 County Durham and Darlington NHS Foundation Trust, Darlington, UK; 4 International Centre for Life, Biosciences Institute, Newcastle University, Newcastle Upon Tyne, NE1 3BZ UK

**Keywords:** Hip fracture, Muscle, Outcomes, Sarcopenia, Systematic review

## Abstract

**Purpose:**

Sarcopenia is diagnosed on the basis of low muscle strength, with low muscle mass used to confirm diagnosis. The added value of measuring muscle mass is unclear. We undertook a systematic review to assess whether muscle mass measurement in patients with hip fracture was acceptable, feasible and independently associated with adverse outcomes.

**Methods:**

Electronic databases (MEDLINE, EMBASE, CENTRAL, CINAHL, Clinicaltrials.gov) were searched for studies of patients with hip fracture aged ≥ 60 with perioperative muscle mass or strength assessments. Associations with postoperative outcomes including death, length of stay and activities of daily living were extracted. Risk-of-bias was assessed using the AXIS and ROBINS-I tools. Due to the degree of study heterogeneity, data were analysed by narrative synthesis.

**Results:**

The search strategy identified 3317 records. 36 studies were included with 7860 participants. Acceptability of muscle mass measurement was not assessed, but measurement appeared feasible using biompedance, dual energy x-ray absorptiometry and computed tomography. Univariate analyses indicated that lower muscle mass was associated with higher death rates at 30 days, worse mobility, worse activity of daily living metrics and worse physical performance but there was no significant association with length of stay or postoperative complications. Four studies included both muscle mass and strength in multivariable analyses; muscle mass was a significant independent predictor of only one adverse outcome in a single study after adjustment for muscle strength and other predictor variables.

**Conclusion:**

Current data suggest that muscle mass assessment offers no additional prognostic information to muscle strength measures in patients with hip fracture.

**Supplementary Information:**

The online version contains supplementary material available at 10.1007/s41999-024-01102-x.

## Key summary points

**Aim:**

We systematically reviewed whether muscle mass measurement was independently associated with adverse outcomes in patients with hip fracture. Our secondary aim was to systematically review whether muscle mass assessment was feasible and acceptable in this cohort.

**Findings:**

There are no studies specifically assessing feasibility and acceptability of muscle mass assessment in patients with hip fracture. Low muscle mass is associated with increased mortality, worse mobility and physical performance in unadjusted analyses.

**Message:**

Included studies suggest muscle mass offers no additional prognostic benefit to muscle strength when assessing sarcopenia in acute hip fracture.

**Supplementary Information:**

The online version contains supplementary material available at 10.1007/s41999-024-01102-x.

## Introduction

Hip fractures are common, costly and life-threatening injuries both in the United Kingdom (UK) and worldwide [1]. Over the last 2 decades, national standardization of hip fracture management has improved quality of care and patient outcomes in the UK [2, 3]. Predicting post-operative outcomes is important when discussing prognosis with patients and family, to enable evidence-based discussions about care [4]. Current validated risk prediction tools in hip fracture include the Nottingham Hip Fracture Score (NHFS) and Almelo Hip Fracture score [5, 6]. Existing scores have moderate discriminative ability for in-hospital, 30- and 120-day mortality and functional outcomes but have only weak predictive ability for outcomes, such as length of stay and postoperative complications [7].

Current hip fracture risk prediction scores do not consider muscle strength and mass, yet sarcopenia (the age-related loss of muscle strength and mass) is known to be a key driver of falls, dependency, and earlier death, and an important component of the physical frailty syndrome [8–10]. Current definitions of sarcopenia require confirmation of low muscle strength, with low muscle mass also being a component of most definitions [11–13]. Measuring muscle strength in clinical practice (for example handgrip strength) is straightforward, inexpensive, and can easily be incorporated into the preoperative assessment of hip fracture [14]. Measuring muscle mass is more complex; simple techniques such as bioelectrical impendence assessment (BIA) are inaccurate and require equations calibrated to specific populations and devices; techniques such as dual X-ray absorptiometry (DXA), cross-sectional imaging via computed tomography (CT), or magnetic resonance imaging (MRI) are more expensive and less accessible. Before muscle mass measurement is considered for incorporation into routine clinical risk assessment for patients with hip fracture, it is important to determine whether these measures are feasible and acceptable for delivery at scale in routine clinical practice, and if they add valuable prognostic value to existing risk scores with or without muscle strength measures.

The aim of this study was therefore to systematically review the available literature to determine whether: (a) muscle mass assessment measures are associated with adverse postoperative outcomes in patients with hip fracture, (b) measuring muscle mass provides additional prognostic value to existing scores that incorporate muscle strength measures in patients with hip fracture and (c) current tools used to assess muscle mass are feasible and acceptable for patients with hip fracture.

## Methods

### Search strategy and selection criteria

A prespecified systematic review protocol was developed prior to commencing the work, and this was published on the PROSPERO online database in July 2021 (Registration number: CRD42021274981). A literature search was performed using the CENTRAL, Ovid MEDLINE, EMBASE, CINAHL and clinicaltrials.gov databases. Citation searching of studies was also undertaken to identify additional studies not detected in database searching. Supplementary Table 1 includes an example search strategy used for Ovid MEDLINE. Searches were conducted from database inception through to the end of November 2023. Studies were deemed eligible for inclusion if (a) a full-text paper was available; (b) the study population was adult hip fracture patients with a mean age of 60 years or above who were hospitalized for surgery; (c) studies included an assessment of muscle mass or strength in hip fracture patients before surgery or during acute hospital admission. Papers were excluded if inclusion criteria were not met.

### Data extraction

3 authors (J.P., S.J. or J.G.) independently reviewed titles and abstracts to determine eligibility for inclusion. Disagreement was discussed between reviewers to determine eligibility. 1 reviewer (J.P.) reviewed all full-text studies with a second reviewer (S.J. or J.G.) independently assessing a random sample of 10% of full-text studies. Data were extracted into a standardized template that included: mean age, gender, location, healthcare setting, reasons for patient exclusion, tool and units used to assess muscle mass/strength, timing of assessment, postoperative outcomes assessed, statistical analysis and adjustments made. For assessment of feasibility and postoperative outcomes, we aimed to compare either the same muscle mass assessment tool as used for the intervention or the currently accepted ‘gold standard’ modalities of CT or MRI. If CT or MRI was unavailable, DXA was employed as an alternative comparison. Prognostic value of muscle mass was assessed by statistical analyses examining the independent contribution of muscle mass, strength and prognostic scores. The full list of tools, muscle mass estimates, and characteristics extracted is summarized in Supplementary Tables 2 and 3.

### Data analysis

Reviewers independently assessed the identified studies for risk of bias, with the second reviewer (S.J. or J.G.) assessing ~ 10% to mitigate errors. Non-randomized studies were assessed using the Cochrane Risk of Bias in Non-randomized studies of Interventions (ROBINS-I) tool [15]. As the review assessed diagnostic testing, domain 2 (bias in selection of participants) was amended accordingly to determine whether patient selection reflected the wider hip fracture population. Studies identified at critical risk of bias were excluded. Cross-sectional studies were assessed using the appraisal tool for Cross-Sectional Studies (AXIS) [16]. As only baseline data was taken from randomized controlled trials, it was decided that the AXIS tool would be appropriate.

Assessment of feasibility and acceptability was determined using the proportion of patients able to complete muscle mass assessment, excluding patients who declined assessment or without data collection. This measure proposed to capture the number of patients who could have had sarcopenia assessed through alternative methods, but due to organizational delay or clinical cause, cognitive impairment and presence of a pacemaker precluding BIA, were not included. Reported effect sizes for the association between muscle mass and a broad range of postoperative outcomes was recorded for both univariate and multivariable analysis.

Meta-analysis was not attempted due to heterogeneity of the populations studied, outcome measures included, and tools used to assess muscle mass or strength. We quantified evidence of an effect using vote counting based on direction of effect in line with synthesis without meta-analysis (SWiM) guidance [17]. We described studies narratively according to their association with adverse outcomes. We described an association as ‘positive’ if lower muscle mass was associated with adverse outcome and ‘negative’ if lower muscle mass was associated with favorable outcome. Positive outcomes in initial univariate analysis were noted, but if subsequent analyses adjusting for covariates found no significance, we deemed the overall outcome as non-significant.

## Results

### Study selection

**Fig. 1:**
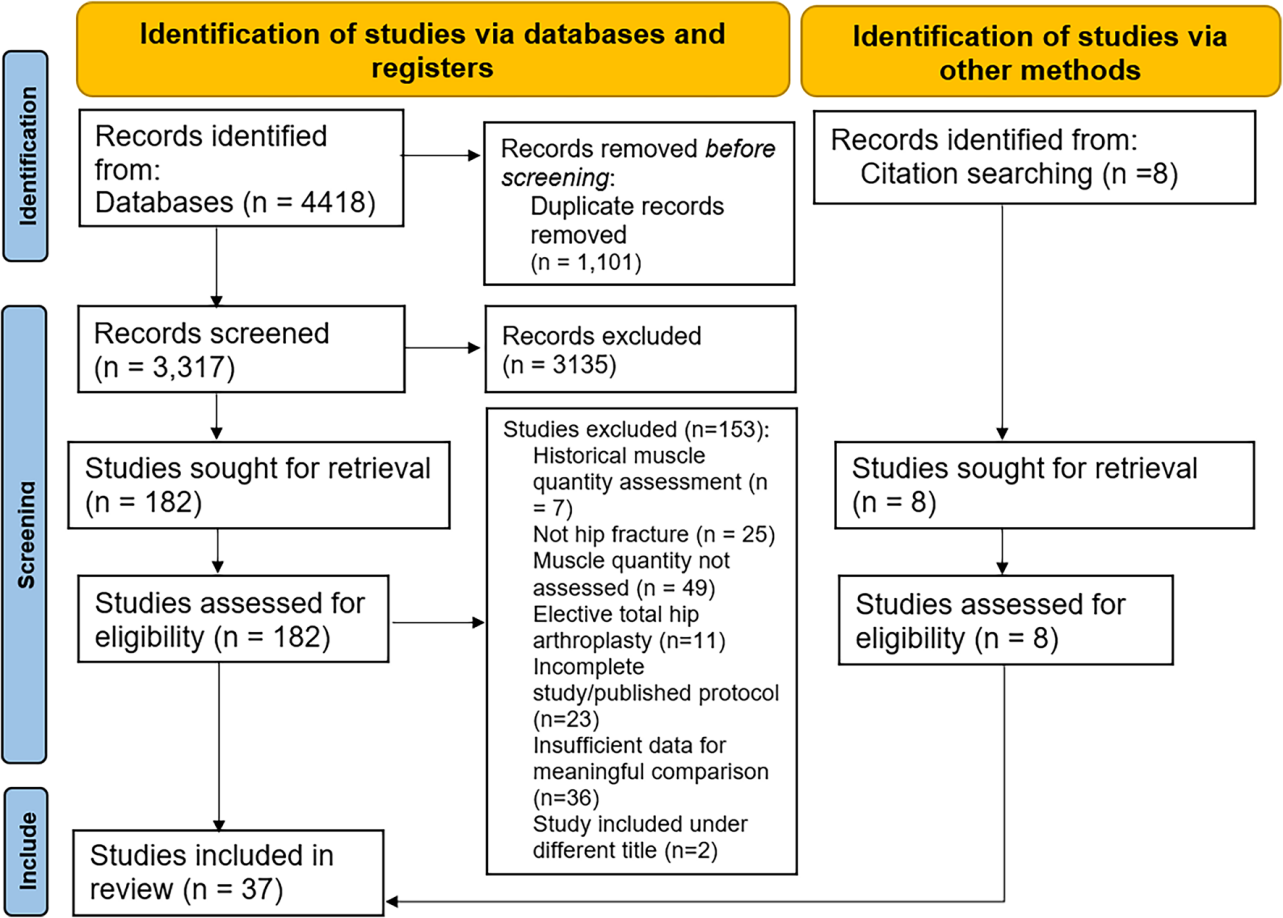
PRISMA flow diagram for selection of studies. Preferred reporting items for systematic reviews and meta-analysis (PRISMA) flow diagram for selection of studies assessing muscle mass, strength feasibility, acceptability, and postoperative outcomes in hip fracture patients

The search identified 3,317 titles, with 182 studies eligible for full-text review. A further 8 studies were included from citation searching during full-text review. A total of 190 full-text studies were reviewed, with 37 studies included in the final analysis. The full selection process and reasons for exclusion are summarized in Fig. 1.

### Study characteristics

The 37 studies reviewed had a variety of designs. 12 were cross-sectional, 24 longitudinal in design (5 case–control and 19 cohort studies) and there was 1 randomized controlled trial with baseline data. Publication dates ranged from 1984 to 2023. Patients were recruited from acute trauma wards (29 studies) and rehabilitation hospitals (8 studies). Study sizes ranged from 40 to 1003 participants, with a total of 7860 participants with a mean age range of75 to 86. Full study details are provided in Supplementary Table 4.

### Risk of *bias*

Of the 25 studies assessed using ROBINS-I, 7 were deemed to be at moderate risk of bias, 16 at serious risk and 2 at critical risk and therefore excluded. Studies at critical risk had inadequate reporting of participant information and missing data [18, 19]. Using AXIS, sample size was not justified in 8/12 there were concerns about non-response bias in 8/12 studies. Full results are displayed in Supplementary Figs. 1 and 2.

### Associations between muscle mass and hip fracture postoperative outcomes

16 studies were included, identifying 31 associations between muscle mass and hip fracture postoperative outcome. There were 7 ‘positive’ associations between lower muscle mass and worse outcomes, and 24 non-significant associations. Findings are summarized in Table 1 and Supplementary Table 5 gives details of each association by assessment tool.

**Table 1:** Summary of direction of association between muscle mass assessment and hip fracture postoperative outcomes

Direction of effect with decreased muscle mass	Postoperative Outcome						Total
	Greater Mortality	Lower Barthel Index	Worse Mobility	Increased length of stay	More perioperative complications	Worse physical performance measures	
Positive association	3	1	2	0	0	1	7
No statistical significance	9	3	1	3	5	3	24
Negative association	0	0	0	0	0	0	0

A positive association was determined if lower muscle mass was associated with adverse outcome

### Mortality

7 cohort studies (*n* = 2663 total patients) contained 17 analyses that assessed the association between muscle mass and mortality [20–26]. 3 analyses showed a significant association between lower muscle mass and higher mortality; 5 unadjusted analyses that showed significant associations became non-significant when analyses were adjusted for other prognostic factors, and 9 analyses did not show significant associations. Full data are reported in Table 2.

**Table 2:** Summary of associations between muscle mass and mortality outcomes alongside direction of association in hip fracture patients

Muscle mass assessment tool	Study	Muscle mass assessment	Mortality outcome	Analysis performed	Strength of association with lower muscle mass	Direction of association
Decreased muscle mass by DXA	Iida, 2021 [[20]]	Low SMI (kg/m^2^)	1-year mortality	Multivariable Cox proportional hazards	HR = 3.2 (1.1–9.3) p = 0.033	Positive
			In-hospital mortality	Student’s t-test	p = 0.17	Not significant
	Kim, 2022 [21]	Low ASM (kg/m^2^)	1-, 2-, 5-year mortality	Chi-squared tests	“Associated” p = not provided	Positive
			Mortality (Unclear timepoint)	Cox proportional hazards	Women HR = 1.3 (0.6–3.1) Men HR = 4.3 (0.5–36.1)	Not significant
Decreased muscle mass by BIA	Menéndez-Colino, 2018 [24]	SMI (kg/m^2^)	1-year mortality	Bivariate Cox proportional hazards, subsequent multivariable analysis	Cox: “Associated” HR = Not provided, p = 0.005 MV analysis: “Dropped out”	Not significant
		Low SMI (kg/m^2^)	1-year mortality	Bivariate Cox Proportional hazards	HR = Not provided p = 0.23	Not significant
	Malafarina,2019 [23]	Low SMI (kg/m^2^)	7-year mortality	Cox proportional hazards	HR = 1.2 (1.0–1.5) p = 0.08	Not significant
	Sanchez-Torralvo, 2023 [51]	Fat free muscle index (kg/m^2^)	3-, 6-, 12-month mortality	Chi-squared test	3 m p = 0.53 6 m p = 0.06 12 m p = 0.06	Not significant
Decreased muscle mass by TSF	Meyer, 2000[25]	Triceps skinfold thickness	3.5-year mortality	Relative risk comparison to control	Lowest quarter: RR = 1.7 (1.1–2.7), Second quarter: RR = 1.9 (1.1–3.0) Third quarter: RR = 1.3 (0.7–2.5) Highest quarter: RR = 1.2 (0.5–3.1)	Positive
Decreased muscle mass by CT	Kim, 2018 [22]	SMI (L3)	1-year mortality	Kaplan–Meier analysis and log-rank test	Mortality rate (MR) Sarcopenic vs non-sarcopenic 22.2 vs 19.6, p = 0.79	Not significant
		SMI (L3)	5-year mortality	Kaplan–Meier analysis and log-rank test	Mortality rate (MR) Sarcopenic vs non-sarcopenic 82.7 vs 52.7, p = 0.03	Positive
		SMI (L3)	5-year mortality	Multivariable Cox analysis using backward selection model	HR: 2.1 (1.1–4.0) p = 0.02	Positive

Summary of associations between muscle mass and mortality outcomes alongside direction of association in hip fracture patients. A positive association was determined if lower muscle mass was associated with adverse outcome at most complex statistical analysis

*DXA* Dual-energy X-ray absorptiometry, *TSF* Triceps skinfold thickness, *BIA* Bioelectrical impedance analysis, *SMI* Skeletal muscle index, *ASM* Appendicular skeletal muscle mass, *HGS* Hand grip strength, *BMI* Body mass index, *SONSQ* The short portable mental status questionnaire, *BMD* Bone mineral density, *ASA* American society of anesthesiologists, *L3*:Third lumbar vertebra *HR* Hazard ratio, *MR* Mortality rate

### Activities of daily living

3 studies, (cohort, case–control and cross-sectional study, *n* = 785 total patients) assessed association using the Barthel Index [20, 27, 28]. 1 study (*n* = 337) found a significant association between low appendicular skeletal muscle index (ASMI) measured using DXA and lower (worse) discharge Barthel Index [20]. The 2 other studies (*n* = 448) performed 3 analyses which did not show an association between DXA-derived ASMI and Barthel Index on discharge after adjustment for other covariates [27, 28].

### Mobility

3 cohort studies (*n* = 304 total patients) assessed association with mobility. 1 study (*n* = 71) reported a significant association between thigh cross-sectional area assessed by CT at 2 months and gait speed at 2 months [29]. This study also noted a significant association between limb cross-sectional area and post-operative improvement in gait speed. Another study (*n* = 143) reported no association between appendicular skeletal muscle index assessed by DXA and improvement in the timed up and go test in multiple linear regression [27]. The final study (*n* = 90) reported no association between muscle mass (kg) assessed by DXA and 12 month recovery in mobility function with scoring assessed by independence/assistance/inability to get in/out of bed, chair rise, walk 10 feet, walk 1 block and climb 5 stairs [30].

### Length of stay in hospital

3 studies (1 case–control, 2 cohort, n = 484 total patients) assessed associations between muscle mess and length of hospital stay. They used appendicular skeletal muscle index measured by DXA, BIA or psoas muscle area at the third lumbar vertebra (L3) level on computed tomography [20, 31, 32]. No significant associations were found between these measures and length of stay.

### Postoperative complications

3 cohort studies (*n* = 448 total patients) assessed association between muscle mass and postoperative complications. 1 study (*n* = 57) assessed association between muscle mass using CT/DXA and Clavien–Dindo grading of surgical complications [33]. The ASMI assessed by DXA, total psoas volume at L3 and psoas area at L3 all showed no association with grade of complication [31]. Another study (*n* = 301) found no association between CT-reported gluteus maximum CSA and risk of a second hip fracture, with a final study finding no association between BIA-assessed ASMI and any postoperative complication. [32, 34].

### Physical performance measures

2 studies (cross-sectional and cohort study, *n* = 487 total patients), assessed association between muscle mass and physical performance. 1 study compared DXA-derived muscle mass and a model of factors influencing physical activity assessed using the Yale Physical Activity Survey [35]. Muscle mass was associated with physical activity in females but not in males. The other study found no associated between BIA-derived SMI and change in motor functional independence measure score at 4 weeks [36].

### Prognostic value of muscle mass when used in addition to muscle strength

7 studies (6 cohort and 1 cross-sectional) measured both muscle mass and muscle strength. Studies are summarized in Supplementary Table 4. 3 studies performed analyses of muscle strength and muscle mass separately rather than combining these measures in a single model [23, 25, 30]. 4 studies reported a multivariable analysis including both muscle mass and strength (*n* = 1139 total patients), with outcomes summarized in Table 3 [24, 27, 35, 36]. For mortality, 1 study (*n* = 509) found that handgrip strength was an independent predictor of 1-year mortality, while muscle mass was non-significant in multivariable analysis [24]. The remaining 2 studies performed separate analysis, with only handgrip strength reaching significance for mortality at 7 years and associated with a greater relative risk at 3.5 years [23, 25]. 2 studies assessed the effect of muscle strength and mass on physical activity measures. 1 study (*n* = 339) found a mixed association for DXA-derived total lean mass and handgrip strength with 2-month physical activity using the Yale Physical Activity Survey. Muscle mass but not strength was independently associated with physical activity for women; the converse was found for men [35]. The other study (*n* = 148) assessed the relationship between BIA-derived SMI, handgrip strength and change in motor functional independence measure (FIM) score at 4 weeks [36]. Higher handgrip was independently associated with improved FIM score, while SMI was not.

**Table 3:** Summary of studies containing assessment of both muscle mass and strength in multivariable analysis

Study	Country	Study type	Assessment Tool	Analysis performed	Variables included in analysis	Outcome under test	Was assessment independently associated	
							Muscle mass	Muscle strength
Di Monaco, 2014 [27]	Italy	Prospective cohort	DXA (ASMI), HGS (kg)	Multiple linear regression	HGS, ASMI, BI on admission, age, # medications, pressure ulcers, # concomitant diseases, infection during stay, days between fracture and assessment included in analysis	BI post-rehabilitation BI effectiveness TUG test performance	No No No	Yes Yes Yes
Menéndez-Colino, 2018 [24]	Spain	Prospective cohort	BIA (SMI) HGS (kg)	Bivariate Cox regression analysis, subsequent multivariable analysis	Low HGS, Sex, Age > 85, Baseline BI ≤ 60, SPMSQ > 3, BMI < 21 kg/m^2^, Heart disease, Vitamin D < 20 ng/ml and PTH ≥ 66 pg/ml, Hb < 12 g/L F < 13 g/L M	1-year mortality	No	Yes
Resnick, 2018 [35]	USA	Cross-sectional	DXA (TLM kg) HGS (kg)	Structural equation modelling. Fit tested with Chi^2^	Model included bone mineral density, TLM, HGS, total body fat, age, cognition, comorbidity, resilience scale, pain	2-month Physical activity (Yale Physical Activity Survey)	Men no Women yes	Men yes Women no
Irisawa, 2022 [36]	Japan	Prospective cohort	BIA (SMI kg/m^2^) HGS (kg)	Multivariable logistic regression	Geriatric nutritional risk index, SMI, HGS, Phase angle	4-week change in Functional Independence measure motor score	No	Yes

*DXA* Dual-energy X-ray absorptiometry, *TSF* Triceps skinfold thickness, *BIA* Bioelectrical impedance analysis, *SMI* Skeletal muscle index, *ASMI* Appendicular skeletal muscle mass index, *TLM* Total lean mass, *TUG* Timed up and go test, *BI* Barthel index, *Hb* Hemoglobin, *SPSMQ* Short portable mental status questionnaire

1 study (*n* = 143) found that ASMI assessed by DXA was determined to be non-significant while handgrip strength was associated with improved change in timed up and go (TUG) test performance [27]. 1 study assessed recovery in mobility by interview using separate analyses[30]. Mobility recovery was not associated with change in muscle mass by DXA, while loss of handgrip strength was associated with worse mobility recovery.

1 study (*n* = 143) assessed Barthel Index after rehabilitation and Barthel ‘effectiveness’ as proportion of potential improvement including both mass and strength [27]. For both analyses, ASMI determined by DXA was non-significant, while handgrip strength was significantly associated with improved outcomes. No studies assessed the association between both muscle mass and strength with increased length of stay or postoperative complications.

### Feasibility and acceptability

Few studies formally assessed feasibility at the time of patient hospitalization; 6 studies did not include any information detailing the reasons for patient exclusion from assessment. Table 4 summarizes the 31 studies assessing muscle mass, the tool used, and the proportion of patients assessed successfully. No papers included data on economic, patient or staff acceptability. Supplementary Table 6 summarizes the proportional completion and reason for patient exclusion for each study.

**Table 4:** Summary of studies assessing muscle mass in older hip fracture patients with sufficient data to assess feasibility

Tool	Sufficient data to assess feasibility	Percentage of patients assessed successfully
Dual-energy X-ray absorptiometry	14	68–92%
Triceps skinfold thickness	3	61–94%
Computed tomography	5	50–93%
Bioelectrical impedance analysis	9	50–95%

Patients were assessed as successful by number of patients able to have muscle mass assessed within total number of patients who did not have assessment due to organizational factors or clinical reasons. We excluded patients who refused assessment or without data collection. Percentage range shows lowest-highest % of patients able to complete muscle mass assessment for each assessment tool for studies included in review

## Discussion

To our knowledge, this is the first systematic review comparing muscle mass and strength assessment in hip fracture. Our results suggest that muscle mass measurement appears feasible (using BIA, DXA and CT), low muscle mass is associated with adverse outcomes in some, but not all studies, and that muscle mass assessment may add little additional prognostic value to assessment of muscle strength. We found that the overall quality of study design and reporting was low, with few studies specifically designed for diagnostic evaluation.

Our review suggests that lower muscle mass is associated with worse postoperative patient outcomes, with more evidence for mortality than for other outcomes. A previous systematic review by Liu et al. reported sarcopenia as a predictor of all-cause mortality in community-dwelling older people, including either muscle mass or strength assessment [9]. A review by Chiang et al. noted an association between sarcopenia and poorer postoperative functional recovery in hip fracture and a further study by Willey et al*.* noted a significant association between serial BIA analysis of skeletal muscle mass and Patient-Reported Outcomes Measurement Information System (PROMIS) physical function domains[32, 37]. We also found evidence that lower muscle mass was also associated with worse activity of daily living metrics, mobility assessment and physical performance measures however, there were conflicting results from studies included. This finding aligns with previous literature on sarcopenia and activities of daily living [38].

We found no associations between low muscle mass and increased length of stay or postoperative complication scores. This is surprising, as in healthy older adults sarcopenia is associated with poor mobility and adverse postoperative outcomes in surgical patients [39, 40]. Other drivers (e.g. susceptibility to infection, organizational factors) may be more directly linked and thus more strongly associated with length of stay than muscle mass. In many of the included studies, there was a lack of adjustment for well-recognized confounders such as cognitive status. The observational studies were mostly deemed to have moderate or serious risk of bias in the domains of confounding, primarily due to the exclusion of medically unwell patients or patients with diagnoses such as dementia. Future research should ideally have a standardized design and produce outputs adjusted for known covariates.

Perhaps most importantly for clinical practice, muscle mass did not appear to add additional prognostic value when muscle strength was also measured. All 4 studies directly comparing muscle strength and mass found muscle strength to be an independent predictor of adverse outcomes, with muscle mass failing to reach statistical significance in 3 studies when included in models already containing muscle strength. These finding support the EWGSOP2 focus on assessment of muscle strength prior to muscle mass in the diagnostic algorithm of sarcopenia. We hypothesize this is because current measures of muscle mass measure lean mass, which is a mixture of muscle and other tissues. Cross-sectional imaging techniques that purport to measure muscle mass may also contain other intramuscular tissues such as fat. It is also possible that muscle strength is influenced by a broader range of factors than muscle mass, potentially including factors that reflect whole-body health (e.g. neurological function, cardiorespiratory function) and that muscle strength therefore provides a more informative measure of integrative whole-body function than muscle mass for prognostic purposes. Furthermore, the EWGSOP2 guidelines highlight numerous studies recognizing muscle strength as being superior to mass in predicting adverse outcomes [41–43]. Given the acceptability, speed, and cost-effective nature of handgrip strength assessment in routine clinical practice, the lack of prognostic value added by measuring muscle mass suggests that the time and effort required to assess muscle mass in patients with hip fracture may not add clinical value [14].

Routine sarcopenia screening in clinical practice may enhance prediction of patient outcomes following hip fracture. This information can be used to estimate and discuss prognosis with patients and to enable evidence-based discussions about inpatient care, as well as to facilitate discharge planning and quantify recovery potential. Most studies included in our review did not report feasibility in a transparent or consistent way, and no studies assessed acceptability. However, some comparable studies have been undertaken with similar patient cohorts. In 2019, Ibrahim et al*.* examined the feasibility of assessing sarcopenia using muscle strength and mass in acutely unwell hospitalized older people. They found assessment of handgrip strength to be feasible, but that assessment of muscle mass presented challenges [42]. A 2013 review by Mijnarends et al*.* determined that BIA was feasible and acceptable in community-dwelling older people, but that further evaluation was required, which aligns with our findings [44]. By comparison, implementation of handgrip strength assessment in hip fracture patients has been shown to be acceptable to staff and cost-effective to implement [14]. We postulate that due to the baseline characteristics of the hip fracture cohort, it can be more challenging to assess feasibility/acceptability. This is likely due to exclusion of patients with cognitive impairment, comorbidities and acute illness from such assessments. This may be important to consider when trying to implement muscle mass and strength assessment in routine clinical practice.

Almost all the studies included in this review assessed muscle mass in the days after admission to hospital for fracture. While assessment of muscle mass in the acute setting may be challenging, hip fractures have been shown to have a substantial negative effect on muscle mass and strength measured after surgery [45]. The lack of studies assessing muscle mass using MRI or ultrasound indicates further research using these tools is necessary to establish their value. It may therefore be more feasible to assess muscle mass by DXA, BIA or CT either during admission or as part of routine perioperative assessment. Sarcopenia assessment in routine practice is part of a suite of outcome prediction biomarkers alongside clinical review of the patient. Alternative methods of sarcopenia assessment such as muscle strength assessment using HGS may be cheaper and easier to deploy in the hip fracture cohort.

Strengths of our review included a best-practice approach when planning, conducting and interpreting a systematic review. We used a prespecified protocol, with a clearly defined clinical question. There was a rigorous approach to assessment of bias using either the ROBINS-I or AXIS tool. A second reviewer was used to mitigate errors at the data-extraction stage. A number of limitations of our review methods also require highlighting. We identified 37 papers and report some key findings, however, our review included a limited evidence base, potentially narrow inclusion criteria and heterogeneity of analyses which precluded meta-analysis of postoperative outcome measures. The broad scope of our literature search may have included older studies, where data collected are no longer relevant due to evolution of clinical practice. It is also possible that the search may have missed some studies, although measures were taken to avoid this. The timing of the sarcopenia assessment may have affected the assessment of muscle mass in the included studies. Furthermore, there was a difference in diagnostic cut-off thresholds between studies. The tools we reviewed are susceptible to gender, ethnicity, and height variations in measurement, and are therefore not a perfect measure of whole-body muscle mass [12].

The studies included in the review used broad exclusion criteria, and therefore excluded patients who could not complete assessment, were acutely unwell, or cognitively impaired. Similarly, no measures were taken to adequately address and categorize non-responders, beyond a reason cited for exclusion from studies. It is recognized that patients with cognitive impairment, multimorbidity and acutely unwell patients are underserved in clinical research[46]. Consequently, it is likely that the included studies are at risk of selection bias and exclusion of the most unwell patients. Due to the lack of analysis about true/false positives or negatives of the included tests, it was not possible for us to produce a summary receiver operating characteristic curve for the feasibility of muscle mass assessment tools.

Our review also highlighted a paucity of studies using measures of muscle mass to assess sarcopenia. EWGSOP2 recommends use of DXA, BIA, CT and MRI in research studies of sarcopenia[12]. Few studies used the modalities of CT, with none for MRI, which may be influenced by the cost, inconvenience, and high clinical demand for these modalities in the acute setting. While publication bias may contribute, the fact that 24 out of 31 of the associations found in this review had no statistical significance indicates this is less likely. The widespread use of CT/MRI imaging for undiagnosed hip fracture patients provides a window for opportunistic assessment of muscle mass for some patients [47]. Further research in a clinical setting is recommended. The small number of studies assessing both muscle mass and strength and their risk of bias (4 serious, 3 moderate) also needs to be considered. Overall, studies performed poorly in the domains of confounding and selection of participants. Analyses performed in individual studies did not control for possible covariates and selected healthier participants. The results may therefore not be representative of the overall hip fracture population.

### Emerging methods of estimating muscle mass

An alternative method of estimating muscle mass, the D_3_-creatine (D_3_-Cr) dilution, has been shown to be a simple, clinically feasible risk predictor of hip fracture in healthy older men [48]. There are currently limited numbers of studies in a clinical setting, with a narrative review by Pagano et al. in 2024 identifying 15 studies correlating D_3_-Cr to clinical outcomes [49]. This study noted a positive correlation between D_3_-Cr and MRI/DXA assessment of muscle mass, with less consistent associations for measures of muscle strength including HGS. Barriers to widespread clinical use include isotope cost, requirements for laboratory analysis and logistics of isotope transport to patients. Furthermore, D_3_-Cr values may be affected by muscle breakdown in hip fracture patients [50]. Nevertheless, ongoing research and future assessment of sarcopenia may include D_3_Cr assessment.

## Conclusion

Although limited by the quality and scope of available literature, our findings suggest that muscle mass measurement offers no additional prognostic benefit to muscle strength when assessing sarcopenia in patients with acute hip fracture. Additional high-quality studies of different muscle mass measurement techniques, specifically designed to evaluate the acceptability, feasibility and prognostic value of muscle mass measures added to muscle strength measures would be desirable. Given that muscle strength assessment is cheaper, easier, and more strongly associated with adverse outcomes, our findings suggest that at present, routine assessment of muscle mass in hip fracture patients has limited value in clinical practice.

## Supplementary Information

Below is the link to the electronic supplementary material.

Supplementary file1 (DOCX 443 KB)

## References

[1] Blankart CR, van Gool K, Papanicolas I, Bernal-Delgado E, Bowden N, Estupiñán-Romero F et al (2021) International comparison of spending and utilization at the end of life for hip fracture patients. Health Serv Res 56(S3):1370–1382 34490633 10.1111/1475-6773.13734 PMC8579204

[2] Middleton M (2018) Orthogeriatrics and hip fracture care in the UK: factors driving change to more integrated models of care. Geriatrics (Basel) 3(3):55 31011092 10.3390/geriatrics3030055 PMC6319212

[3] Physicians RCo. The challenge of the next decade: are hip fracture services ready? A review of data from the National Hip Fracture Database (January–December 2019). London: RCP; 2021.

[4] Stubbs TA, Doherty WJ, Chaplin A, Langford S, Reed MR, Sayer AA et al (2023) Using pre-fracture mobility to augment prediction of post-operative outcomes in hip fracture. Eur Geriatr Med 14(2):285–293 37002428 10.1007/s41999-023-00767-0 PMC10113355

[5] Nijmeijer WS, Folbert EC, Vermeer M, Slaets JP, Hegeman JH (2016) Prediction of early mortality following hip fracture surgery in frail elderly: the almelo hip fracture score (AHFS). Injury 47(10):2138–2143 27469403 10.1016/j.injury.2016.07.022

[6] Wiles MD, Moran CG, Sahota O, Moppett IK (2011) Nottingham Hip fracture score as a predictor of one year mortality in patients undergoing surgical repair of fractured neck of femur. Br J Anaesth 106(4):501–504 21278153 10.1093/bja/aeq405

[7] Doherty WJ, Stubbs TA, Chaplin A, Reed MR, Sayer AA, Witham MD et al (2021) Prediction of postoperative outcomes following hip fracture surgery: independent validation and recalibration of the nottingham hip fracture score. J Am Med Dir Assoc 22(3):663–9.e2 32893139 10.1016/j.jamda.2020.07.013

[8] Cruz-Jentoft AJ, Sayer AA (2019) Sarcopenia. Lancet 393(10191):2636–2646 31171417 10.1016/S0140-6736(19)31138-9

[9] Liu P, Hao Q, Hai S, Wang H, Cao L, Dong B (2017) Sarcopenia as a predictor of all-cause mortality among community-dwelling older people: a systematic review and meta-analysis. Maturitas 103:16–22 28778327 10.1016/j.maturitas.2017.04.007

[10] Yeung SSY, Reijnierse EM, Pham VK, Trappenburg MC, Lim WK, Meskers CGM et al (2019) Sarcopenia and its association with falls and fractures in older adults: A systematic review and meta-analysis. J Cachexia Sarcopenia Muscle 10(3):485–500 30993881 10.1002/jcsm.12411 PMC6596401

[11] Chen LK, Woo J, Assantachai P, Auyeung TW, Chou MY, Iijima K et al (2020) Asian working group for sarcopenia: 2019 consensus update on sarcopenia diagnosis and treatment. J Am Med Dir Assoc 21(3):300–7.e2 32033882 10.1016/j.jamda.2019.12.012

[12] Cruz-Jentoft AJ, Bahat G, Bauer J, Boirie Y, Bruyère O, Cederholm T et al (2019) Sarcopenia: revised european consensus on definition and diagnosis. Age Ageing 48(1):16–31 30312372 10.1093/ageing/afy169 PMC6322506

[13] Studenski SA, Peters KW, Alley DE, Cawthon PM, McLean RR, Harris TB et al (2014) The FNIH sarcopenia project: rationale, study description, conference recommendations, and final estimates. J Gerontol A Biol Sci Med Sci 69(5):547–558 24737557 10.1093/gerona/glu010 PMC3991146

[14] Doherty WJ, Stubbs TA, Chaplin A, Langford S, Sinclair N, Ibrahim K et al (2021) Implementing grip strength assessment in hip fracture patients: a feasibility project. J Frailty Sarcopenia Falls 6(2):66–78 34131603 10.22540/JFSF-06-066 PMC8173531

[15] Sterne JA, Hernán MA, Reeves BC, Savović J, Berkman ND, Viswanathan M et al (2016) ROBINS-I: a tool for assessing risk of bias in non-randomised studies of interventions. BMJ 355:i4919 27733354 10.1136/bmj.i4919 PMC5062054

[16] Downes MJ, Brennan ML, Williams HC, Dean RS (2016) Development of a critical appraisal tool to assess the quality of cross-sectional studies (AXIS). BMJ Open 6(12):e011458 27932337 10.1136/bmjopen-2016-011458 PMC5168618

[17] Campbell M, McKenzie JE, Sowden A, Katikireddi SV, Brennan SE, Ellis S et al (2020) Synthesis without meta-analysis (SWiM) in systematic reviews: reporting guideline. BMJ 368:l6890 31948937 10.1136/bmj.l6890 PMC7190266

[18] Bean N, Bennett KM, Lehmann AB (1995) Habitus and hip fracture revisited: skeletal size, strength and cognition rather than thinness? Age Ageing 24(6):481–484 8588536 10.1093/ageing/24.6.481

[19] Wehren LE, Hawkes WG, Hebel JR, Orwig DL, Magaziner J (2005) Bone mineral density, soft tissue body composition, strength, and functioning after hip fracture. J Gerontol: Series A 60(1):80–84 10.1093/gerona/60.1.80 15741287

[20] Iida H, Seki T, Sakai Y, Watanabe T, Wakao N, Matsui H et al (2021) Low muscle mass affect hip fracture treatment outcomes in older individuals: a single-institution case-control study. BMC Musculoskelet Disord. 10.1186/s12891-021-04143-6 33750363 10.1186/s12891-021-04143-6 PMC7945055

[21] Kim HS, Park JW, Lee YK, Yoo JI, Choi YS, Yoon BH et al (2022) Prevalence of sarcopenia and mortality rate in older adults with hip fracture. J Am Geriatr Soc 70(8):2379–2385 35657018 10.1111/jgs.17905

[22] Kim YK, Yi SR, Lee YH, Kwon J, Jang SI, Park SH (2018) Effect of sarcopenia on postoperative mortality in osteoporotic hip fracture patients. J Bone Metab 25(4):227–233 30574467 10.11005/jbm.2018.25.4.227 PMC6288605

[23] Malafarina V, Malafarina C, Biain Ugarte A, Martinez JA, Abete Goni I, Zulet MA (2019) Factors associated with sarcopenia and 7-year mortality in very old patients with hip fracture admitted to rehabilitation units: a pragmatic study. Nutrients 11(9):18 10.3390/nu11092243 PMC6770746 31540409

[24] Menéndez-Colino R, Alarcon T, Gotor P, Queipo R, Ramírez-Martín R, Otero A et al (2018) Baseline and pre-operative 1-year mortality risk factors in a cohort of 509 hip fracture patients consecutively admitted to a co-managed orthogeriatric unit (FONDA Cohort). Injury 49(3):656–661 29329713 10.1016/j.injury.2018.01.003

[25] Meyer HE, Tverdal A, Falch JA, Pedersen JI (2000) Factors associated with mortality after hip fracture. Osteoporos Int 11(3):228–232 10824238 10.1007/s001980050285

[26] Sanchez-Castellano C, Martin-Aragon S, Vaquero-Pinto N, Bermejo-Bescos P, Merello de Miguel A, Cruz-Jentoft AJ (2019) Prevalence of sarcopenia and characteristics of sarcopenic subjects in patients over 80 years with hip fracture. Nutr Hosp 36(4):813–818 31282168 10.20960/nh.02607

[27] Di Monaco M, Castiglioni C, De Toma E, Gardin L, Giordano S, Di Monaco R et al (2014) Handgrip strength but not appendicular lean mass is an independent predictor of functional outcome in hip-fracture women: a short-term prospective study. Arch Phys Med Rehabil 95(9):1719–1724 24769122 10.1016/j.apmr.2014.04.003

[28] Di Monaco M, Castiglioni C, Vallero F, Di Monaco R, Tappero R (2011) Appendicular lean mass does not mediate the significant association between vitamin D status and functional outcome in hip-fracture women. Arch Phys Med Rehabil 92(2):271–276 21272724 10.1016/j.apmr.2010.09.028

[29] Eastlack M, Miller RR, Hicks GE, Gruber-Baldini A, Orwig DL, Magaziner J et al (2022) Thigh Muscle Composition and Its Relationship to Functional Recovery Post Hip Fracture Over Time and Between Sexes. J Gerontol A Biol Sci Med Sci 77(12):2445–2452 35580856 10.1093/gerona/glac112 PMC9799201

[30] Visser M, Harris TB, Fox KM, Hawkes W, Hebel JR, Yahiro JY et al (2000) Change in muscle mass and muscle strength after a hip fracture: relationship to mobility recovery. J Gerontol A Biol Sci Med Sci 55(8):M434–M440 10952365 10.1093/gerona/55.8.m434

[31] So SP, Lee BS, Kim JW (2021) Psoas muscle volume as an opportunistic diagnostic tool to assess sarcopenia in patients with hip fractures: A retrospective cohort study. J Personal Med. 10.3390/jpm11121338 10.3390/jpm11121338 PMC8709037 34945811

[32] Willey MC, Owen EC, Miller A, Glass N, Kirkpatrick T, Fitzpatrick D et al (2023) Substantial loss of skeletal muscle mass occurs after femoral fragility fracture. J Bone Joint Surg Am 105(22):1777–1785 37738373 10.2106/JBJS.23.00353

[33] Dindo D, Demartines N, Clavien PA (2004) Classification of surgical complications: a new proposal with evaluation in a cohort of 6336 patients and results of a survey. Ann Surg 240(2):205–213 15273542 10.1097/01.sla.0000133083.54934.ae PMC1360123

[34] Wang L, Yin L, Yang M, Ge Y, Liu Y, Su Y et al (2022) Muscle density is an independent risk factor of second hip fracture: a prospective cohort study. J Cachexia Sarcopenia Muscle 13(3):1927–1937 35429146 10.1002/jcsm.12996 PMC9178374

[35] Resnick B, Hebel JR, Gruber-Baldini AL, Hicks GE, Hochberg MC, Orwig D et al (2018) The impact of body composition, pain and resilience on physical activity, physical function and physical performance at 2 months post hip fracture. Arch Gerontol Geriatr 76:34–40 29455057 10.1016/j.archger.2018.01.010 PMC5882522

[36] Irisawa H, Mizushima T (2022) Relationship between nutritional status, body composition, muscle strength, and functional recovery in patients with proximal femur fracture. Nutrients 14(11):2298 35684096 10.3390/nu14112298 PMC9183158

[37] Chiang M-H, Kuo Y-J, Chen Y-P (2021) The association between sarcopenia and postoperative outcomes among older adults with hip fracture: a systematic review. J Appl Gerontol 40(12):1903–1913 33870747 10.1177/07334648211006519

[38] Wang DXM, Yao J, Zirek Y, Reijnierse EM, Maier AB (2020) Muscle mass, strength, and physical performance predicting activities of daily living: a meta-analysis. J Cachexia Sarcopenia Muscle 11(1):3–25 31788969 10.1002/jcsm.12502 PMC7015244

[39] Morley JE, Abbatecola AM, Argiles JM, Baracos V, Bauer J, Bhasin S et al (2011) Sarcopenia with limited mobility: an international consensus. J Am Med Dir Assoc 12(6):403–409 21640657 10.1016/j.jamda.2011.04.014 PMC5100674

[40] Trejo-Avila M, Bozada-Gutiérrez K, Valenzuela-Salazar C, Herrera-Esquivel J, Moreno-Portillo M (2021) Sarcopenia predicts worse postoperative outcomes and decreased survival rates in patients with colorectal cancer: a systematic review and meta-analysis. Int J Colorectal Dis 36(6):1077–1096 33481108 10.1007/s00384-021-03839-4

[41] Mitchell WK, Williams J, Atherton P, Larvin M, Lund J, Narici M (2012) Sarcopenia, dynapenia, and the impact of advancing age on human skeletal muscle size and strength; a quantitative review. Front Physiol 3:260 22934016 10.3389/fphys.2012.00260 PMC3429036

[42] Ibrahim K, Howson FFA, Culliford DJ, Sayer AA, Roberts HC (2019) The feasibility of assessing frailty and sarcopenia in hospitalised older people: a comparison of commonly used tools. BMC Geriatr 19(1):42 30770722 10.1186/s12877-019-1053-y PMC6377779

[43] Schaap LA, van Schoor NM, Lips P, Visser M (2018) Associations of sarcopenia definitions, and their components, with the incidence of recurrent falling and fractures: the longitudinal aging study amsterdam. J Gerontol A Biol Sci Med Sci 73(9):1199–1204 29300839 10.1093/gerona/glx245

[44] Mijnarends DM, Meijers JM, Halfens RJ, ter Borg S, Luiking YC, Verlaan S et al (2013) Validity and reliability of tools to measure muscle mass, strength, and physical performance in community-dwelling older people: a systematic review. J Am Med Dir Assoc 14(3):170–178 23276432 10.1016/j.jamda.2012.10.009

[45] Reider L, Owen EC, Dreyer HC, Fitton LS, Willey MC, M. (2023) Loss of muscle mass and strength after hip fracture: an intervention target for nutrition supplementation. Current Osteoporosis Rep 21(6):710–718 10.1007/s11914-023-00836-0 38019345

[46] NIHR. Improving inclusion of under-served groups in clinical research: Guidance from the NIHR-INCLUDE project. UK:NIHR2020 [Available from: www.nihr.ac.uk/documents/improving-inclusion-of-under-served-groups-in-clinical-research-guidance-from-include-project/25435

[47] Centre NCG (2011) The management of hip fracture in adults. National Clinical Guideline Centre, London

[48] Cawthon PM, Peters KE, Cummings SR, Orwoll ES, Hoffman AR, Ensrud KE et al (2022) Association between muscle mass determined by d3-creatine dilution and incident fractures in a prospective cohort study of older men. J Bone Miner Res 37(7):1213–1220 35253257 10.1002/jbmr.4505 PMC9283198

[49] Pagano AP, Montenegro J, Oliveira CLP, Desai N, Gonzalez MC, Cawthon PM et al (2023) Estimating muscle mass using d3-creatine dilution: a narrative review of clinical implications and comparison with other methods. J Gerontol: Series A. 10.1093/gerona/glad280 10.1093/gerona/glad280 PMC10959434 38135279

[50] Hedström M, Ljungqvist O, Cederholm T (2006) Metabolism and catabolism in hip fracture patients: nutritional and anabolic intervention–a review. Acta Orthop 77(5):741–747 17068704 10.1080/17453670610012926

[51] Sanchez-Torralvo FJ, Perez-del-Rio V, Garcia-Olivares M, Porras N, Abuin-Fernandez J, Bravo-Bardaji MF et al (2023) Global subjective assessment and mini nutritional assessment short form better predict mortality than glim malnutrition criteria in elderly patients with hip fracture. Nutrients 15(8):1828 37111046 10.3390/nu15081828 PMC10140871

