# Supplementary material for: Feasibility, acceptability and prognostic value of muscle mass and strength measurement in patients with hip fracture: a systematic review
Source: Eur Geriatr Med. 2024 Nov 29;15(6):1603–14. doi: 10.1007/s41999-024-01102-x (PMC11632060; doi:10.1007/s41999-024-01102-x)
Supplement: Supplementary file 1 — Supplementary file1 (DOCX 443 KB) [file 41999_2024_1102_MOESM1_ESM.docx]

**Article Title: Feasibility, acceptability and prognostic value of muscle mass and strength measurement in patients with hip fracture: a systematic review**

**Supplementary Material**

**Journal Name: European Geriatric Medicine**

**Author** **Names; affiliation and email address of corresponding authors**

James Prowse^1,2^

Sharlene Jaiswal^1,2^

Jack Gentle^3^

Antony K Sorial*^4^

Miles D Witham*^1,2^

1. AGE Research Group, Translational and Clinical Research Institute, Faculty of Medical Sciences, Newcastle University, Newcastle upon Tyne, UK

2. NIHR Newcastle Biomedical Research Centre, Newcastle upon Tyne NHS Foundation Trust, Cumbria Northumberland Tyne and Wear NHS Foundation Trust and Newcastle University, Newcastle upon Tyne, UK

3. County Durham and Darlington NHS Foundation Trust, Darlington, UK

4^.^ Biosciences Institute, Newcastle University, International Centre for Life, Newcastle upon Tyne, NE1 3BZ, UK.

Antony K Sorial* and Miles D Witham* are joint senior authors.

Correspondence to:

Mr Antony K Sorial, Biosciences Institute, Newcastle University, International Centre for Life, Newcastle upon Tyne, NE1 3BZ, UK.

Tel: +44 191 241 8626. Email: [Tony.Sorial@newcastle.ac.uk](mailto:Tony.Sorial@newcastle.ac.uk). Twitter: @tsorial

Professor Miles Witham, AGE Research Group, NIHR Newcastle Biomedical Research Centre, Campus for Ageing and Vitality, Newcastle upon Tyne, NE4 5PL, UK.

Tel: +44 191 208 1317. Email: [Miles.Witham@newcastle.ac.uk](mailto:Miles.Witham@newcastle.ac.uk). Twitter: @OlderTrialsProf

**Contents**

**Supplementary Table 1**: Search strategy used for Ovid MEDLINE(R) ALL (1946 to 30/11/23).

**Supplementary Table 2**: Summary of tools used to assess muscle mass, adjustments, and possible variations.

**Supplementary Table 3**: Summary of study characteristics extracted

**Supplementary Table 4**: Summary of Included Studies

**Supplementary Table 5**: Summary of muscle mass assessment tool, number of studies utilising tool to assess muscle mass and number of associations reported.

**Supplementary Table 6**. Proportion of each study completing muscle mass assessments and reason for patient exclusion

**Supplementary Figure 1**. Summary of risk of bias domains using risk of bias in non-randomised studies of interventions (ROBINS-I) for observational studies included within the review.

**Supplementary Figure 2.** Summary of risk-of-bias domains for cross-sectional studies included in the review assessed using the appraisal tool for cross-sectional studies (AXIS)

**Appendix 1**. Full reference list

**Supplementary Table 1: Search strategy used for Ovid MEDLINE(R) ALL (1946 to 30/11/23).**

| Step | Keyword searched | Results |
| --- | --- | --- |
| 1 | Exp Hip fracture/  [mp=title, abstract, heading word, table of contents, key concepts, original title, tests & measures] | 29444 |
| 2 | Hip fracture.mp | 14812 |
| 3 | Hip surg*.mp | 3491 |
| 4 | Hip opera*.mp | 202 |
| 5 | 1 or 2 or 3 or 4 | 32917 |
| 6 | Exp Muscle, Skeletal/ | 306615 |
| 7 | Exp Muscle weakness/ | 9789 |
| 8 | Exp Muscle strength/ | 45819 |
| 9 | Exp Sarcopenia/ | 9956 |
| 10 | Muscle mass.mp | 22348 |
| 11 | grip strength.mp | 11963 |
| 12 | Muscle volume.mp | 1961 |
| 13 | Muscle quantity.mp | 225 |
| 14 | exp Postoperative Complications/ | 618488 |
| 15 | exp Prognosis/ | 1947076 |
| 16 | 6 or 7 or 8 or 9 or 10 or 11 or 12 or 13 or 14 or 15 | 2753007 |
| 17 | exp dual x ray absorptiometry/ | 26210 |
| 18 | bioelectrical impedance analysis.mp | 4050 |
| 19 | exp Magnetic Resonance Imaging/ | 541134 |
| 20 | computed tomography.mp | 296014 |
| 21 | exp Anthropometry/ | 566350 |
| 22 | ultrasound.mp | 266875 |
| 23 | 17 or 18 or 19 or 20 or 21 or 22 | 1591459 |
| 24 | 5 AND 16 AND 23 | 659 |

| **Supplementary Table 2****: Summary of tools used to assess muscle mass, adjustments, and possible variations.** | | |
| --- | --- | --- |
| **Assessment Tool** | **Methods of muscle mass estimation** | **Notes** |
| Dual-energy X-ray absorptiometry | Comparison of two X-ray beams with different attenuations. Prediction equations used to compute lean soft tissue mass of all 4 limbs (ASM). | ASM can be adjusted for body size by division using height^2^, body mass index or weight. Influenced by hydration status |
| Bioelectrical impedance analysis | Estimates SMM or ASM based on whole-body electrical conductivity and application of prediction equation. | Multiple prediction equations relevant to population derived from. Affected by age, sex, ethnicity. Can be influenced by hydration status. SMM/ASM by BIA can also be adjusted for body size. |
| Computed tomography | Computes series of X-ray images taken from different angles to create cross-sectional images of the body. Estimation from specific plane e.g. third lumbar cross-sectional muscle area or total body. | Cut-off points for low muscle mass not well defined. |
| Magnetic Resonance Imaging | Magnetic fields and radio waves computed to generate cross-sectional image of body. Muscle cross-section estimation taken from specific plane or total body. | Cut-off points for low muscle mass not well defined. |
| Anthropometry | Physical measurement of individual’s size used to reflect nutritional status. E.g. Thickness of triceps skinfold | Variation dependent on individual and population environment. |
| *ASM: Appendicular Skeletal Muscle Mass, SMM: Skeletal Muscle Mass BIA: Bioelectrical Impedance Analysis* | | |

**Supplementary Table 3: Summary of study characteristics extracted**

Study information and characteristics of included study

- Paper ID (Internal Numbering), Paper Title
- Author and publication location
- Type of paper
- Location of study
- Inclusion/Exclusion Criteria
- Study population characteristics
- Muscle mass or strength assessment tool used
- Outcome measurements and associated effect size
- Timing of muscle mass assessment relative to surgery
- Level of formal support
- Additional Notes

Muscle mass assessment and feasibility (Aim 1)

*The same process was completed for intervention and comparator (if relevant)*

- Number of patients Successful
- Number of patients unsuccessful
- Timing relative to surgery
- Notes
- Number of patients screened, eligible, agreed, excluded
- Reasons for exclusion/dropout

Muscle mass and comparator outcomes (Aim 2 and 3)

*The same process was completed for intervention and comparator (if relevant)*

- How muscle mass/strength measured
- Adjustments in regression analysis/covariates
- Muscle mass value
- Muscle strength value
- Outcome measurement (Effect size, covariates, univariate/multivariate analysis)
- Additional Notes

**Supplementary Table 4: Summary of Included Studies**

| First Author and Publication Year | Country of study | Study Design | Healthcare Setting | Sample size | Average age (years ± standard deviation unless otherwise stated) | Muscle mass/strength assessment modality | Timing of assessment post fracture | Number Completed and reason for exclusion | Postoperative Outcomes Included | Effect Size | Note |
| --- | --- | --- | --- | --- | --- | --- | --- | --- | --- | --- | --- |
| Bachrach-Lindstrom, 2001[52] | Sweden | Case-control | Hospital | 88 (44I, 44C) | 84.2±5.0 C,  84.2±5.1 I | TSF (cm), MAC (cm), BIA (Lean mass kg) | 4-6d | 88/95  3 Withdrew, 4 Death  3 Refused | N/A | N/A | MAC used to calculate Arm muscle circumference |
| Bean, 1995[18] | UK | Prospective Case-control | Hospital | 50F | 79.5±8.4 | HGS (kg) | <72h admission | Not stated | Hip fracture incidence | HGS Multiple regression R=0.59 R^2^ =0.35 p<0.0001 | Critical risk of bias due to missing data. |
| Cervera-Diaz, 2023[53] | Spain | Cross-sectional | Hospital | 152F  34M | 86.2 (range 82-90) | HGS (kg), BIA (ALMI/h^2^) | <48h admission | 186/274  Emergency surgery 38  Assessed >48h 50  ‘Did not meet criteria’  60  Did not agree 40 | N/A | N/A | Exclusion criteria included underlying disease with <6 month life expectancy, persistent oedema, prior nutritional supplementation, cognitive impairment, pacemaker or prosthesis, patient unable to walk, non-acceptance |
| Cha, 2022[54] | Korea | Retrospective cohort | Hospital | 129F  51M | M sarcopenic 75.6±9.5  Non sarcopenia 74.3±9.0  F  Sarcopenic 78.1±8.1  Non  Sarcopenic 76.2±9.85 | HGS (kg)  DXA (ASMI) | Not stated | DXA 180/289  67 no DXA, 20 ‘mental health issues’ e.g. dementia/depression | Braden Scale at admission (pressure ulcer) | χ2-test relationship with quartile of HGS/ASMI for M and F  M patients Braden scale and HGS no association (p=0.251) F patients Braden scale associated (p=0.041)  M patients Braden Scale and SMI associated (p=0.02)  F patients Braden Scale and SMI not associated (p=0.304) | Used AWGS – required both low muscle strength and mass  Mixed association between tools by gender  Braden score at admission, so not included. |
| Cheng, 2020[55] | Taiwan | Cross-sectional | Hospital | 103F 36M | 80.7 ± 9.7 | DXA (RASM) | 137.04±154.67h postadmission | 139/154  16 major trauma  4 hip/knee arthroplasty  8 acute diseases | Not studied | N/A | Same population as above study |
| Di Monaco, 2021[56] | Italy | Cross-sectional | Rehabilitation hospital | 183F | 80.0±7.7 | DXA (ASMI), HGS (kg) |  | DXA 183/199  4 could not complete, 4 pathological fracture, 8 acute disease  1 refuse  HGS: 183/199  As above. | BI at discharge | N/A | Not possible to infer the association of muscle mass or strength as mixed classification of sarcopenia. |
| Di Monaco, 2014[27] | Italy | Prospective cohort | Rehabilitation hospital | 143F | 79.2±7.4 | DXA(ASMI) HGS (kg) | 21.1±8.7d post fracture | DXA=123/148  15 could not walk 6 pathological fracture 2 hip/knee arthroplasty, 2 acute disease  1 refusal  HGS 123/148  As above | Barthel index after rehabilitation, Barthel index effectiveness (proportion of potential improvement), TUG test performance | Multiple linear regression model BI after rehabilitation HGS Partial r=0.22 p=0.016 ASMI Partial r=0.01 p=0.92  BI effectiveness HGS partial r=0.23 p=0.011 ASMI=0.05 p=0.62  TUG Test performance HGS Partial r=-0.25 p=0.005 ASMI=0.09 p=0.30 | Both HGS and ASMI included in multivariate model. |
| Di Monaco, 2011[28] | Italy | Cross-sectional | Rehabilitation hospital | 280F | 79.7±7.4 | DXA (ASMI) | 21.2±6.2d | 280/305  14 major trauma/cancer  4 hip/knee arthroplasty, 7 Pathological Fracture/ pacemaker/ death | Barthel Index at end of inpatient stay | Correlation of aLM/ht^2 and postrehabilitation BI (p=0.04, P=0.94) and Vit D (p=0.025 P=0.682) | aLM/ht^2 does not mediate association between vitamin D and functional outcome |
| Di Monaco, 2012[57] | Italy | Cross-sectional | Rehabilitation hospital | 531F 60M | 80.0±7.4 F 81.4±7.5M | DXA (ASMI) | Interval from fracture 18.3±8.8F 19.4±8.2M | 591/620  25 major trauma, 4 hip/knee arthroplasty  10 refuse | N/A | N/A | This study analysed prevalence of sarcopenia in hip fracture patients. As this was not an outcome for the review it was not analysed. |
| Di Monaco, 2007[58] | Italy | Cross-sectional | Rehabilitation hospital | 327F | 79.5 ± 7.5 |  | 22.1±7.5d post-fracture | 299/327  16 pathological fracture  4 hip/knee arthroplasty,  8 refusal/  death/  Acute disease | Not studied | N/A |  |
| Diaz de Bustamante  2018[59] | Spain | Cross-sectional | Hospital | 403F 106M | 85.6 ± 6.9 | BIA (ASMI)  HGS (Kg) | <72h admission | BIA: 479/505  26 died or lost to follow up  HGS: 479/505 | N/A | N/A | Study did not separate analysis of muscle mass/strength but grouped as sarcopenia. |
| Eastlack, 2022[29] | USA | Prospective cohort | Hospital | 34F  37M | F 80.3±7.0  M 79.0±7.6 | CT (Thigh muscle CSA) | 2 month postoperatively | N/A | 2 month and 6 month Gait speed, Chair rise, SPPB | One-way ANOVA between tertiles alongside posthoc testing for tertile pairs.  Gait speed p≤ .005  Chair rise p≤ .001  SPPB p ≤ .008 | Also reviewed muscle attenuation (quality).  CSA adjusted for sex and muscle CSA differences. Also compared CSA difference between injured/non-injured limb. |
| González-Montalvo, 2016[60] | Spain | Prospective cohort | Hospital | 479 | 85.3 ± 6.8 | BIA (ASMI), HGS (kg) | <72h admission | BIA 479/509  30 “various reasons: refusal, died before assess, surgery before assess”  HGS 438/509  Previous 30, 41 could not complete HGS | BI lost at discharge | N/A | Patients in this study were diagnosed as sarcopenic according to EWGSOP criteria. Analysis used the dependent variable of sarcopenia. It was therefore not possible to separate the effects or prognostic ability of muscle strength or mass. |
| Groenendijik, 2020[61] | Netherlands | Cross-sectional | Rehabilitation ward | 29F 11M | 81.6 ± 8.0 | BIA (ASMM), HGS (kg)  Ultrasound (Rectus femoris thickness mm) | Median 4d (range 2-10d) postadmission | BIA 37/41  3 pathological fracture 1 acute disease  HGS 36/41  3 fracture, 1 actute disease, 1 unable  3 refused | Not studied | N/A | Ultrasound measurements not used further. |
| Hicks, 2019[62] | USA | Cross-sectional | Hospital | 22F 19M | 79.4±4.7F 79.8±5.0M | CT (L4-5 Trunk Muscle area cm^2^) | <2m post fracture | 41/67  26 withdrew  149 refused | N/A | N/A | Study primarily looks at difference in CSA between populations. No outcomes relevant to study so excluded. |
| Hida, 2013[63] | Japan | Cross-sectional | Hospital | 304F 53M | 82.7±9.3 F, 80.3±9.4M | DXA (A  SMI) | <48h admission | 357/391  34 urgent repair  31 refused | Not studied | N/A | Lower appendicular SMI (P<0.001) and higher prevalence of sarcopenia (P<0.001) in Hip Fx group. Presence of sarcopenia associated with occurrence of hip fracture in stepwise logistic regression analysis B=0.389 OR=1.476(1.154-1.888) p=0.002 |
| Hoekstra, 2011[64] | Netherlands | Controlled Prospective cohort | Hospital | 48F 13M I  48F 18M C | 80.6±7.2 | BIA (Body cell mass kg) | “During hospitalisation” | 127/152  13 major trauma, 12 pacemaker,  25 refused  1 language | Not studied | N/A | N/A |
| Iida, 2021[20] | Japan | Case-Control | Hospital | 270F 67M | Low MM (n=231): 83.4±7.1F 82.4±7.0M, Normal MM(n=106) 85.0±6.9F, 80.5±8.1M p=0.242 students | DXA (ASMI) for Low MM (AWGS) | “Upon admission” | 337/381  44 hip/knee arthroplasty  45 no data collected on admisison | Hospital mortality, length of stay(d), hospital discharge rate, 1y mortality Barthel index at discharge, 1y mortality | Student t test hospital mortality p=0.173, LOS p=0.811, BI(at discharge)=<0.001, home discharge p=0.035  1y mortality Cox proportional hazards Low muscle mass HR=3.192(1.097-9.226) p=0.033 | Values used for hospital mortality are small |
| Irisawa, 2022[36] | Japan | Prospective cohort | Rehabilitation hospital | 121 F  27M | 84.1±7.8 | HGS (kg)  BIA (SMI) | Mean admission 22d post fracture. | Both 148/184  Pacemaker 2  High functional independence measure score 10  Cognitive impairment 15  Dysphasia 1  Early discharge 8  Unclear 9 | Motor Functional Indepence Measure (FIM) score change from admission vs 4w | Univariate analysis (FIM)  High muscle strength OR 1.34(0.65-2.76) p=0.43  High SMI OR=0.86(0.42-1.77) p=0.68  Multivariate analysis (ADL)  Muscle strength  male: r = 0.41  female r=0.43  Muscle quantity  Male r = -0.03  Female r= -0.01 or -0.02 (believe authors confused figures in text) | Also assessed muscle quality using phase angle.  Adjustment for sex.  AWGS cut-off used.  High muscle strength M<26.0 kg  F< 18.0 kg  High SMI  M<7.0kg/m2  F <5.7 kg/m2 |
| Jung, 2022[65] | South Korea | Retrospective cohort | Hospital | Intertrochanter 19F 6M  Femoral Neck 17F 8M | IT 79.8±6.9  FN 79.5±6.8 | CT various Muscular cross-sectional area | 1-3 years postop | 50/90  Bilateral operation 19  Unable to ambulate 10  Neuromuscular disease 4  Hemiparesis after CVA 7 | N/A | N/A | Only comparison of changes in muscle mass pre/postoperatively.  Excluded from analysis as focused on postop CT analysis. |
| Kim, 2022[21] | South Korea | Retrospective cohort | Hospital | 760 F  243 M | F 74.9 ± 10.2  M 78.9 ± 9.8 | HGS (kg)  DXA (ASM/height^2^ , ASM/weight, ASM/BMI) | “Preoperatively” | DXA 1003/1203  Unavailable before operation 148  Cog impairment 52  51 refuse  HGS unclear | 1,2,5 year mortality | Univariate analysis Mcnemar’s test  1,2,5 year mortality all discriminated by ASM/height^2^  Mixed picture for ASM/wt and ASM/BMI depending on gender and #years.  Multivariate analysis  Cox proportional hazards  1,2,5 year mortality no significance but did not include HGS | Cox adjusted for age, diagnosis, height, weight, ASM and comorbidity.  Both HGS and ASM used to classify sarcopenia using AWGS.  Proportional regression analysis in supplementary material. No timepoint |
| Kim, 2018[22] | Korea | Retrospective cohort | Hospital | 64F 27M | Sarcopenia 81.9±5.8  Non-sarcopenia 75.3±4.9 | CT (SMI at L3) | “Within 1 year of hip surgery” | 91/117  10 could not complete DXA  11 bilateral hip fracture  5 osteoporotic | 1y mortality rate, 5y mortality rate, 5 year mortality | 1y mortality Kaplin-Meier analysis for survival curve and log-rank tests Sarcopenia MR=22.2(22.1-22.3) Non-sarcopenia MR=19.6 (19.5-19.7) p=0.793  5y mortality Kaplin-Meier analysis for survival curve and log-rank tests Sarcopenia MR=82.7(82.6-82.8) Non-sarcopenia MR=52.7 (52.6-52.8) p=0.028  5y effect on overall mortality Multivariable Cox analysis using backward selection model Sarcopenia HR=2.144(1.140-4.031) p=0.018 | Defined sarcopenic patients as low SMI at L3.  Age, gender, sarcopenia, BMD, ASA grade, fracture location, operation type include in multivariate analysis |
| Lau 1993[66] | China | Case-control | Hospital | 131F 32M | 79±8 F 76±8M | Iliac/Tricep skinfold thickness (mm) and HGS (Kg) | <1w admission | Not stated | Relative risk of FN fracture for quartiles of grip strength and iliac skinfold by multiple logistic regression | Men relative risk FN Iliac skinfold RR= 1.1(0.7-1.6) grip strength RR=1.8(1.2-2.9)  Women relative risk FN Iliac skinfold RR=2.1(1.7-2.8) HGS=1.1(0.9-1.4) | Both HGS and skinfold included in regression. “Results of multiple regression were similar when bicep or tricep skinfold thickness was used instead of iliac” |
| Malafarina, 2019[23] | Spain | Cohort | Hospital | 138F 49M | 85.2±6.3 | BIA(ASMI), HGS (kg) | “Upon admission” | BIA: 187/206  2 hip/knee arthroplasty 2 major trauma  10 death in hospital 5 acute disease  HGS 187/206 | 7-year mortality | Low HGS Cox cumulative survival HR=1.76(1.08-2.88) p=0.024  Low Muscle mass Cox 7 year cumulative survival HR=1.20(0.98-1.47) p=0.076 | Also assessment of HGS/ASMI as independent factors contributing to incident/chronic sarcopenia. Not relevant to review. Muscle mass and strength not simultaneously in analysis. |
| Malafarina, 2017[67] | Spain | RCT | Rehabilitation hospital | I=49 C=43 | I=85.7±6.5  C=84.7±6.3 | HGS, BIA (aLM) | <72h admission | 94/109  15 withdrew  22 “Other reasons  67 “Not met inclusion criteria” | None | Student t test comparing difference between values on discharge vs admission: HGS p=0.752  aLM by Sergi et al p=0.020 | Comparison between control and intervention groups. Not includes in analysis of outcomes. |
| Menendez-Colino, 2018[24] | Spain | Prospective cohort | Hospital | 403F 106M | 85.65±6.9 | BIA (Muscle mass index) | <72h admission | 482/509  No explanation for non-inclusion. | 1y mortality | Bivariate cox regression analysis: Muscle mass index (kg/m2) Survivors=8.26 (7.2-9.7) non-survivors =9.05 (7.71-10.4) p=0.005, Low muscle mass index(kg/m2) Survivors= 74 (19%) Non-survivors=15 (12.7%) p=0.230  Multivariate analysis Low HGS (HGS <23kgM <13kg F ) HR=2.088(1.172-3.178) p=0.012 | Muscle mass index dropped out in subsequent multivariate analysis.  HGS independent mortality risk factor |
| Meyer, 2000[25] | Norway | Prospective cohort | Hospital | 198F 50M | 78.4 ± 8.8 | HGS, TSF | <14d admission | HGS 238/473  225 excluded  9 further excluded  TSF 247/472  225 excluded due to nursing home, low cognition. High energy trauma, reduced consciousness death during study follow-up, bone cancer  36 refused | 3.5y mortality | HGS Lower half of distribution Cox proportional hazards RR = 2.30(1.55-3.42)  Upper half RR=0.90 (0.52-1.54)  TSF Lowest quarter RR=1.73 (1.09-2.73), Second quarter 1.87(1.14-3.04) Third quarter 1.32(0.70-2.48) | Strength and muscle mass not both included in a multivariate analysis. No analysis of MAC. Controlled for age, sex. No associated p values. |
| Miller, 2015[68] | USA | Prospective cohort | Hospital | 21F 26M | 80.4±Unclear (Range 65-96) | CT (Thigh cross-sectional) area | <2m | 47/50  3 Withdrew  17 Refused | N/A | N/A | N/A |
| Park, 2022[69] | South Korea | Cross-sectional | Hospital | Unclear-not exact topic paper covered | Unclear-not exact topic paper covered | HGS (kg)  DXA (aSMI) | “preoperatively” | DXA 789/1159  370 no DXA before operation  HGS unclear | N/A | N/A | Study compared changes from time of operation vs 1y follow-up and so demographic data not present |
| Resnick, 2018[35] | USA | Cross-sectional | Hospital | 171F 168M | 81.0 ± 7.7F 80.4±7.7M | DXA (Lean total mass kg), HGS (kg) | <15d admission | DXA 339/362  HGS 339/362  6 ineligible  5 no data  12 informed consent | 2-month Physical activity (PA), Lower extremity gains score (LEGS) and SPPB. | Structural equation modelling. Fit tested with Chi^2^ statistic.  In men Total Lean Mass PA Parameter estimate=-0.049 p*=0.332 LEGS Parameter=-0.171 p*=0.004 SPPB parameter=-0.037 p*=0.382  In Women total lean mass PA Parameter =-0.148 P*=0.051 LEGS Parameter=-0.223 p*=0.013 SPPB Parameter=-0.396 p*=0.001 | Both Lean mass and HGS included in model. (*p ≤ .055). Conssitent association in women but not in men |
| Sanchez-Torralvo, 2023 [51] | Spain | Prospective cohort | Hospital | 238F  62M | 82.9±7.1 | HGS (kg)  BIA (Fat free mass index),  Calf circumference,  Triceps skinfold (TSF) | Not stated | No clear data | 3m, 6m, 12m mortality | Univariate analysis  Chi^2^ analysis  HGS associated with mortality p=0.005, p<0.001, p<0.001 respectively  No significance BIA, calf circum, TSF  None included in multivariate analysis. HGS excluded due to mortality of n=1 in normal HGS patients. | Low HGS classified as bottom 5%. |
| So, 2021[31] | Korea | Retrospective cohort | Hospital | 57F | Mean 79.5 Range 59-93 | DXA (ASMI), HGS (kg) , CT (Total Psoas volume/ area at L3) | “During patient hospital stay” | DXA/CT 57/61  3 hip/knee arthroplasty  1 pathological fracture  1 insufficient data  HGS 46/61  As above  11 could not complete | Grade of postoperative complication (Clavien-dindo), Length of stay, ASA grade, Preoperative walking ability (Koval score) | ASMI and Grade of complication Spearman correlation coefficient =-0.028 p=0.84 Length of stay coefficient=-0.049 p=0.84 ASA grade coefficient=0.057 p=0.67 Koval score coefficient=-0.028 p=0.84  Total psoas volume at L3 and Grade of complication Spearman correlation coefficient=-0.047 p=0.73 Length of stay correlation=-0.056 p=0.71 ASA grade coefficient=-0.062 p=0.65 Koval score=-0.013 p=0.92  Psoas area at L3 and grade of complications spearman correlation coefficient =-0.043 p=0.75 Length of stay coefficient=0.034 p=0.82 ASA grade coefficient=0.032 p=0.81 Koval score coefficient=-0.091 p=0.50 | HGS data was collected, but not used in analysis of postoperative complications |
| Visser, 2000[30] | USA | Prospective cohort | Hospital | 90F | 79.4 (No SD provided) | DXA (Muscle mass kg), HGS (kg) | 3d postadmission or 10d | DXA 90/90  HGS 71/90  19 unable to complete | Recovery in mobility function by interview as assessed by structured interview (Giving points 0-2 for 5 ADL total 10. Change at 12m calculated) | ANOVA Mobility function recovery not related to change in skeletal muscle mass of nonfractured leg or arms.  ANOVA Loss of HGS lead to worse mobility recovery. Mean loss of -28.7% p<0.05 versus tertile III | Data organised by tertile of muscle mass change. No simultaneous analysis of mass and strength. |
| Wang 2022[34] | China | Prospective cohort | Hospital | 193F  108M | 74.6±9.9 | CT (Gluteus maximus cross-sectional area cm^2^) | <48h admission | Not clearly stated | Risk of second fracture (median 4.5y) | Cox proportional hazards  HR= 1.32(0.91-1.91. p=0.14 | Adjusted for age, sex, diabetes, parker mobility score |
| Wehren, 2005[19] | USA | Prospective cohort | Hospital | 205F | 81.0±7.8 | DXA (Lean mass kg), HGS (Kg) | 2d after admission or 10d after admission | Not stated | ADL 2,6,12m post fracture | DXA: Baseline lean mass correlation coefficient =~0 (No p value), HGS:Baseline correlation coefficient=-0.37 (No p) | Critical level of bias , therefore excluded from analysis. |
| Willey, 2023 [32] | USA | Prospective cohort | Hospital | 67F  54M | 77.6±9.0 | BIA (ASMI) | <72h after admission | Not stated | Length of stay (days), any complication | LOS- p=0.678  Complication- p=0.876 |  |
| Yoo, 2016[70] | Korea | Case-control | Hospital | 278F 87M | 78.3±9.8F 63.3±8.7M | DXA (ASMI) | “Preoperatively” | 359/424  34 urgent repair 31 cognitively impaired | Not studied | N/A | In multivariate analysis (general linear model) sarcopenia OR=6.52(4.67-9.09) associated with occurrence of hip fracture  Student’s t test Lower ASMI (p<0.01), leg muscle mass (p<0.001), prevalence of sarcopenia (p<0.001) observed in hip fracture group |

**Supplementary Table 5: Summary of muscle mass assessment tool, number of studies utilising tool to assess muscle mass and number of associations reported.**

| **Tool** | **Number of studies** | **Number of associations reported in studies** |
| --- | --- | --- |
| DXA | 6 | 18 |
| CT | 3 | 6 |
| DXA and CT | 1 | 3 |
| TSF | 1 | 1 |
| MRI | 0 | 0 |
| Ultrasound Scan | 0 | 0 |
| BIA | 5 | 6 |
| **Total** | **17** | **36** |
| *DXA: Dual-energy X-ray absorptiometry CT: Computed tomography TSF: Triceps skinfold thickness MRI: Magnetic resonance Imaging BIA: Bioelectrical impedance analysis* | | |

For five analyses included above, positive associations on univariate analysis were found to become non-significant on multivariable testing when muscle strength measures were also included. See comment on this topic in the text of the paper results section.

**Supplementary Table 6. Proportion of each study completing muscle mass assessments and reason for patient exclusion**

| Study | Completion | Proportion Completed | Reason for exclusion |  |
| --- | --- | --- | --- | --- |
| DXA | | | |  |
| Cheng (2020) | 139/154 | 0.90 | 16 major trauma  4 hip/knee arthroplasty  8 acute diseases |  |
| Di Monaco (2021) | 183/199 | 0.92 | 4 could not complete, 4 pathological fracture, 8 acute disease |  |
| Di Monaco (2014) | 123/148 | 0.83 | 15 unable to walk, 6 pathological fracture, 2 hip/knee arthroplasty, 2 acute disease |  |
| Di Monaco (2011) | 280/305 | 0.92 | 14 major trauma/cancer  4 hip/knee arthroplasty, 7 Pathological Fracture/ pacemaker/ death |  |
| Di Monaco (2012) | 591/620 | 0.95 | 25 major trauma, 4 hip/knee arthroplasty |  |
| Di Monaco (2007) | 299/327 | 0.91 | 16 pathological fracture  4 hip/knee arthroplasty,  8 refusal/death/Acute disease |  |
| Hida (2013) | 357/391 | 0.91 | 34 urgent repair |  |
| Iida (2021) | 337/381 | 0.88 | 44 hip/knee arthroplasty |  |
| Kim (2022) | 1003/1203 | 0.83 | 148 unavailable before operation, 52 Cognitive impairment  51 refused |  |
| Park (2022) | 789/1159 | 0.68 | 370 no DXA before operation |  |
| Yoo (2016) | 359/424 | 0.85 | 34 urgent repair 31 cognitively impaired |  |
| Resnick (2018) | 339/362 | 0.94 | 12 cognitively impaired  6 ineligible, 5 missing data, |  |
| So (2021) | 57/61 | 0.93 | 3 hip/knee arthroplasty, 1 pathological fracture |  |
| Visser (2000) | 90/90 | 1.00 | N/A |  |
| CT | | | |  |
| Hicks (2019) | 41/67 | 0.61 | 26 withdrew during study | |
| Jung (2022) | 50/90 | 0.56 | 19 Bilateral op 19 unable to ambulate, 10 neuromuscular disease, 4 hemiparesis after CVA | |
| Keun Kim (2018) | 91/117 | 0.78 | 10 could not complete DXA (comparison technique for this study), 11 bilateral hip fracture, 5 osteoporotic | |
| Miller (2015) | 47/50 | 0.94 | 3 Withdrew during study | |
| So (2021) | 57/61 | 0.93 | 3 hip/knee arthroplasty,  1 pathological fracture | |
| Triceps skinfold thickness | | | | |
| Bachrach-Lindstrom (2001) | 88/95 | 0.93 | 3 withdrew during study, 4 death | |
| Meyer (2000) | 247/472 | 0.52 | 225 excluded due to nursing home, low cognition. High energy trauma, reduced consciousness death during study follow-up, bone cancer | |
| Meyer (1995) | 246/489 | 0.50 | 117 nursing home, 16 reduced consciousness, 12 quick discharge, 54 cognitive impaired, 6 death during follow-up, 32 pathological fracture, 7 could not complete | |
| Bioimpedance assessment (BIA) | | | | |
| Bachrach-Lindstrom (2001) | 88/95 | 0.79 | 3 withdrew during study, 4 death | |
| Cervera-Diaz (2023) | 186/374 | 0.50 | 38 Emergency surgery, 50 >48h assessment, 60 “did not meet criteria” (common comorbidities)  40 Did not agree | |
| Diaz de Bustamante (2018) | 479/505 | 0.95 | 26 died or lost to follow up | |
| Gonzalez-Montalvo (2016) | 479/509 | 0.94 | various reasons: refusal, died before assess, surgery before asses | |
| Groenendijik (2020) | 37/41 | 0.88 | 3 pathological fracture 1 acute disease | |
| Hoekstra (2011) | 127/152 | 0.84 | 13 major trauma, 12 pacemaker | |
| Irisawa (2022) | 148/184 | 0.80 | 2 pacemaker, 10 high functional independence measure score, 15 cognitive impairment, 1 dysphasia, 8 early discharge  9 unclear | |
| Malafarina (2019) | 187/206 | 0.91 | 2 hip/knee arthroplasty 2 major trauma  10 death in hospital 5 acute disease | |
| Malafarina (2017) | 94/109 | 0.86 | 15 withdrew during study | |


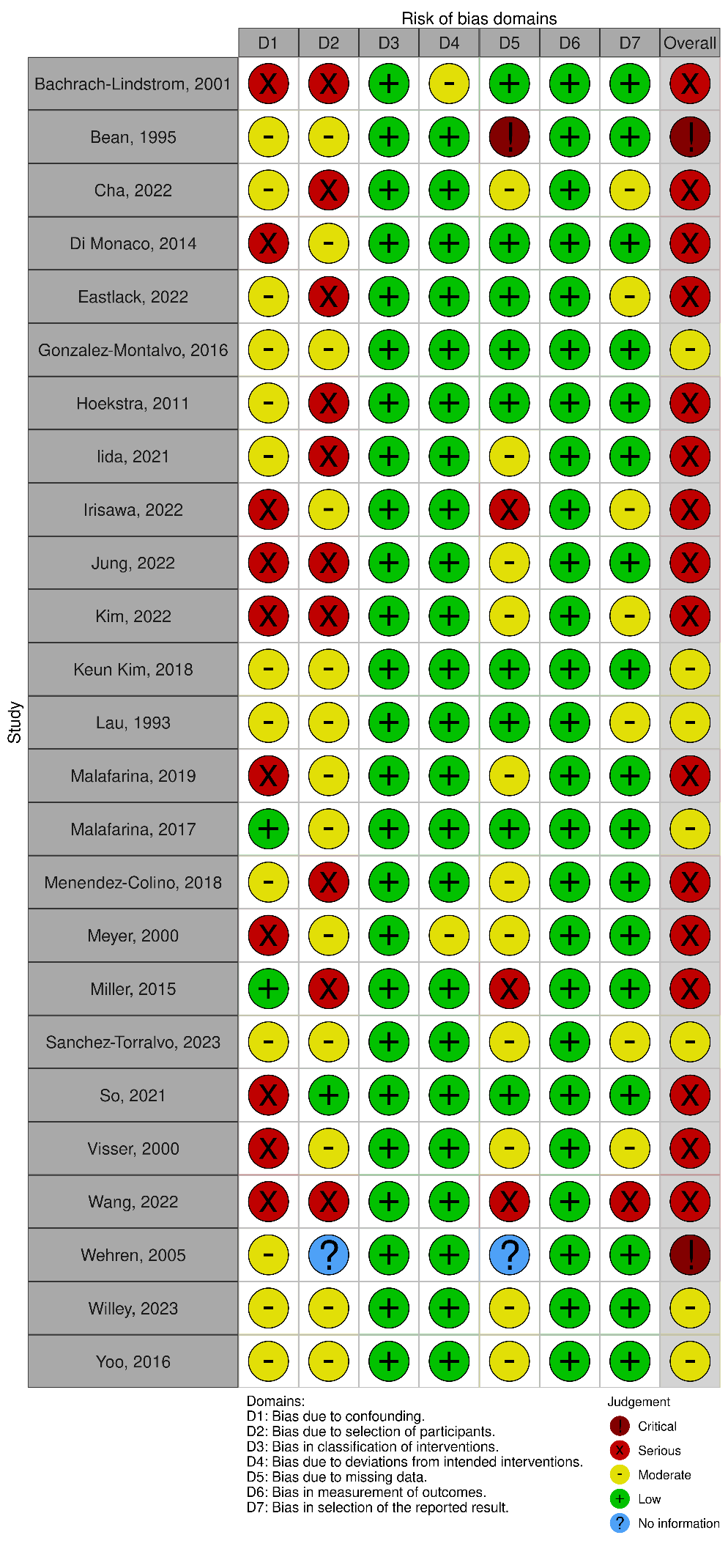
**Supplementary Figure 1. Summary of risk of bias domains using risk of bias in non-randomised studies of interventions (ROBINS-I) for observational studies included within the review. Created using *robvis* [66]**

**Supplementary Figure 2: Summary of risk-of-bias domains for cross-sectional studies included in the review assessed using the appraisal tool for cross-sectional studies (AXIS)**

NB: The assessment refers to the population of hip fracture patients included in each study. Studies were awarded a Y if the question was answered yes, a N if question answered no and NR=Not reported. Red shading has been used to indicate higher risk of bias, green for lower risk of bias and yellow if no response was discernible.

| Question | | Cervera-Diaz, 2023 | Cheng, 2020 | Di Monaco, 2021 | Di Monaco, 2011 | Di Monaco, 2012 | Di Monaco, 2007 | Diaz de Bustamante, 2018 | Groenendijk, 2020 | Hicks, 2019 | Hida, 2012 | Park, 2022 | Resnick, 2018 |
| --- | --- | --- | --- | --- | --- | --- | --- | --- | --- | --- | --- | --- | --- |
| 1 | Were the aims/objectives of the study clear? |  |  |  |  |  |  |  |  |  |  |  |  |
| 2 | Was the study design appropriate for the stated aim(s)? |  |  |  |  |  |  |  |  |  |  |  |  |
| 3 | Was the sample size justified? |  |  |  |  |  |  |  |  |  |  |  |  |
| 4 | Was the target/reference population clearly defined (is it clear who the research was about?) |  |  |  |  |  |  |  |  |  |  |  |  |
| 5 | Was the sample frame taken from an appropriate population base so that it closely represented the target/reference population under investigation |  |  |  |  |  |  |  |  |  |  |  |  |
| 6 | Was the selection process likely to select subjects/participants that were representative of the target/reference population under investigation? |  |  |  |  |  |  |  |  |  |  |  |  |
| 7 | Were measures undertaken to address and categorise non-responders? |  |  |  |  |  |  |  |  |  |  |  |  |
| 8 | Were the risk factor and outcome variables measured appropriate to the aims of the study? |  |  |  |  |  |  |  |  |  |  |  |  |
| 9 | Were the risk factor and outcome variables measured correctly using instruments/measurements that had been trialled, piloted or published previously? |  |  |  |  |  |  |  |  |  |  |  |  |
| 10 | Is it clear what was used to determine statistical significance and/or precision estimates (eg. P-values, confidence intervals)? |  |  |  |  |  |  |  |  |  |  |  |  |
| 11 | Were the methods (including statistical methods) sufficiently described to enable them to be repeated? |  |  |  |  |  |  |  |  |  |  |  |  |
| 12 | Were the basic data adequately described? |  |  |  |  |  |  |  |  |  |  |  |  |
| 13 | Does the response rate raise concerns about non-response bias? |  |  |  |  |  |  |  |  |  |  |  |  |
| 14 | If appropriate, was information about non-responders described |  |  |  |  |  |  |  |  |  |  |  |  |
| 15 | Were the results internally consistent |  |  |  |  |  |  |  |  |  |  |  |  |
| 16 | Were the results presented for all the analyses described in the methods? |  |  |  |  |  |  |  |  |  |  |  |  |
| 17 | Were the author’s discussions and conclusions justified by the results? |  |  |  |  |  |  |  |  |  |  |  |  |
| 18 | Were the limitations of the study discussed? |  |  |  |  |  |  |  |  |  |  |  |  |
| 19 | Were there any funding sources or conflicts of interests that may affect the author’s interpretation of results |  |  |  |  |  |  |  |  |  |  |  |  |
| 20 | Was ethical approval or consent of participants attained? |  |  |  |  |  |  |  |  |  |  |  |  |

**Appendix 1. Full reference list**

1. Blankart CR, van Gool K, Papanicolas I, Bernal-Delgado E, Bowden N, Estupiñán-Romero F, et al. International comparison of spending and utilization at the end of life for hip fracture patients. Health Services Research. 2021;56(S3):1370-82.

2. Middleton M. Orthogeriatrics and Hip Fracture Care in the UK: Factors Driving Change to More Integrated Models of Care. Geriatrics (Basel). 2018;3(3).

3. Physicians RCo. The challenge of the next decade: are hip fracture services ready? A review of data from the National Hip Fracture Database (January–December 2019). London: RCP; 2021.

4. Stubbs TA, Doherty WJ, Chaplin A, Langford S, Reed MR, Sayer AA, et al. Using pre-fracture mobility to augment prediction of post-operative outcomes in hip fracture. Eur Geriatr Med. 2023;14(2):285-93.

5. Nijmeijer WS, Folbert EC, Vermeer M, Slaets JP, Hegeman JH. Prediction of early mortality following hip fracture surgery in frail elderly: The Almelo Hip Fracture Score (AHFS). Injury. 2016;47(10):2138-43.

6. Wiles MD, Moran CG, Sahota O, Moppett IK. Nottingham Hip Fracture Score as a predictor of one year mortality in patients undergoing surgical repair of fractured neck of femur. British Journal of Anaesthesia. 2011;106(4):501-4.

7. Doherty WJ, Stubbs TA, Chaplin A, Reed MR, Sayer AA, Witham MD, et al. Prediction of Postoperative Outcomes Following Hip Fracture Surgery: Independent Validation and Recalibration of the Nottingham Hip Fracture Score. J Am Med Dir Assoc. 2021;22(3):663-9.e2.

8. Cruz-Jentoft AJ, Sayer AA. Sarcopenia. Lancet. 2019;393(10191):2636-46.

9. Liu P, Hao Q, Hai S, Wang H, Cao L, Dong B. Sarcopenia as a predictor of all-cause mortality among community-dwelling older people: A systematic review and meta-analysis. Maturitas. 2017;103:16-22.

10. Yeung SSY, Reijnierse EM, Pham VK, Trappenburg MC, Lim WK, Meskers CGM, et al. Sarcopenia and its association with falls and fractures in older adults: A systematic review and meta-analysis. J Cachexia Sarcopenia Muscle. 2019;10(3):485-500.

11. Chen LK, Woo J, Assantachai P, Auyeung TW, Chou MY, Iijima K, et al. Asian Working Group for Sarcopenia: 2019 Consensus Update on Sarcopenia Diagnosis and Treatment. J Am Med Dir Assoc. 2020;21(3):300-7.e2.

12. Cruz-Jentoft AJ, Bahat G, Bauer J, Boirie Y, Bruyère O, Cederholm T, et al. Sarcopenia: revised European consensus on definition and diagnosis. Age Ageing. 2019;48(1):16-31.

13. Studenski SA, Peters KW, Alley DE, Cawthon PM, McLean RR, Harris TB, et al. The FNIH sarcopenia project: rationale, study description, conference recommendations, and final estimates. J Gerontol A Biol Sci Med Sci. 2014;69(5):547-58.

14. Doherty WJ, Stubbs TA, Chaplin A, Langford S, Sinclair N, Ibrahim K, et al. Implementing grip strength assessment in hip fracture patients: a feasibility project. J Frailty Sarcopenia Falls. 2021;6(2):66-78.

15. Sterne JA, Hernán MA, Reeves BC, Savović J, Berkman ND, Viswanathan M, et al. ROBINS-I: a tool for assessing risk of bias in non-randomised studies of interventions. BMJ. 2016;355:i4919.

16. Downes MJ, Brennan ML, Williams HC, Dean RS. Development of a critical appraisal tool to assess the quality of cross-sectional studies (AXIS). BMJ Open. 2016;6(12):e011458.

17. Campbell M, McKenzie JE, Sowden A, Katikireddi SV, Brennan SE, Ellis S, et al. Synthesis without meta-analysis (SWiM) in systematic reviews: reporting guideline. Bmj. 2020;368:l6890.

18. Bean N, Bennett KM, Lehmann AB. Habitus and hip fracture revisited: skeletal size, strength and cognition rather than thinness? Age & Ageing. 1995;24(6):481-4.

19. Wehren LE, Hawkes WG, Hebel JR, Orwig DL, Magaziner J. Bone Mineral Density, Soft Tissue Body Composition, Strength, and Functioning After Hip Fracture. The Journals of Gerontology: Series A. 2005;60(1):80-4.

20. Iida H, Seki T, Sakai Y, Watanabe T, Wakao N, Matsui H, et al. Low muscle mass affect hip fracture treatment outcomes in older individuals: a single-institution case-control study. BMC Musculoskeletal Disorders. 2021;22(1).

21. Kim HS, Park JW, Lee YK, Yoo JI, Choi YS, Yoon BH, et al. Prevalence of sarcopenia and mortality rate in older adults with hip fracture. Journal of the American Geriatrics Society. 2022;70(8):2379-85.

22. Kim YK, Yi SR, Lee YH, Kwon J, Jang SI, Park SH. Effect of Sarcopenia on Postoperative Mortality in Osteoporotic Hip Fracture Patients. J Bone Metab. 2018;25(4):227-33.

23. Malafarina V, Malafarina C, Biain Ugarte A, Martinez JA, Abete Goni I, Zulet MA. Factors Associated with Sarcopenia and 7-Year Mortality in Very Old Patients with Hip Fracture Admitted to Rehabilitation Units: A Pragmatic Study. Nutrients. 2019;11(9):18.

24. Menéndez-Colino R, Alarcon T, Gotor P, Queipo R, Ramírez-Martín R, Otero A, et al. Baseline and pre-operative 1-year mortality risk factors in a cohort of 509 hip fracture patients consecutively admitted to a co-managed orthogeriatric unit (FONDA Cohort). Injury. 2018;49(3):656-61.

25. Meyer HE, Tverdal A, Falch JA, Pedersen JI. Factors associated with mortality after hip fracture. Osteoporosis International. 2000;11(3):228-32.

26. Sanchez-Castellano C, Martin-Aragon S, Vaquero-Pinto N, Bermejo-Bescos P, Merello de Miguel A, Cruz-Jentoft AJ. [Prevalence of sarcopenia and characteristics of sarcopenic subjects in patients over 80 years with hip fracture]. Nutricion Hospitalaria. 2019;36(4):813-8.

27. Di Monaco M, Castiglioni C, De Toma E, Gardin L, Giordano S, Di Monaco R, et al. Handgrip strength but not appendicular lean mass is an independent predictor of functional outcome in hip-fracture women: a short-term prospective study. Arch Phys Med Rehabil. 2014;95(9):1719-24.

28. Di Monaco M, Castiglioni C, Vallero F, Di Monaco R, Tappero R. Appendicular lean mass does not mediate the significant association between vitamin D status and functional outcome in hip-fracture women. Archives of Physical Medicine & Rehabilitation. 2011;92(2):271-6.

29. Eastlack M, Miller RR, Hicks GE, Gruber-Baldini A, Orwig DL, Magaziner J, et al. Thigh Muscle Composition and Its Relationship to Functional Recovery Post Hip Fracture Over Time and Between Sexes. The journals of gerontology Series A, Biological sciences and medical sciences. 2022;77(12):2445-52.

30. Visser M, Harris TB, Fox KM, Hawkes W, Hebel JR, Yahiro JY, et al. Change in muscle mass and muscle strength after a hip fracture: relationship to mobility recovery. J Gerontol A Biol Sci Med Sci. 2000;55(8):M434-40.

31. So SP, Lee BS, Kim JW. Psoas muscle volume as an opportunistic diagnostic tool to assess sarcopenia in patients with hip fractures: A retrospective cohort study. Journal of Personalized Medicine. 2021;11(12) (no pagination).

32. Willey MC, Owen EC, Miller A, Glass N, Kirkpatrick T, Fitzpatrick D, et al. Substantial Loss of Skeletal Muscle Mass Occurs After Femoral Fragility Fracture. J Bone Joint Surg Am. 2023;105(22):1777-85.

33. Dindo D, Demartines N, Clavien PA. Classification of surgical complications: a new proposal with evaluation in a cohort of 6336 patients and results of a survey. Ann Surg. 2004;240(2):205-13.

34. Wang L, Yin L, Yang M, Ge Y, Liu Y, Su Y, et al. Muscle density is an independent risk factor of second hip fracture: a prospective cohort study. Journal of Cachexia, Sarcopenia and Muscle. 2022;13(3):1927-37.

35. Resnick B, Hebel JR, Gruber-Baldini AL, Hicks GE, Hochberg MC, Orwig D, et al. The impact of body composition, pain and resilience on physical activity, physical function and physical performance at 2 months post hip fracture. Archives of Gerontology & Geriatrics. 2018;76:34-40.

36. Irisawa H, Mizushima T. Relationship between Nutritional Status, Body Composition, Muscle Strength, and Functional Recovery in Patients with Proximal Femur Fracture. Nutrients. 2022;14(11):2298.

37. Chiang M-H, Kuo Y-J, Chen Y-P. The Association Between Sarcopenia and Postoperative Outcomes Among Older Adults With Hip Fracture: A Systematic Review. Journal of Applied Gerontology. 2021;40(12):1903-13.

38. Wang DXM, Yao J, Zirek Y, Reijnierse EM, Maier AB. Muscle mass, strength, and physical performance predicting activities of daily living: a meta-analysis. Journal of Cachexia, Sarcopenia and Muscle. 2020;11(1):3-25.

39. Morley JE, Abbatecola AM, Argiles JM, Baracos V, Bauer J, Bhasin S, et al. Sarcopenia with limited mobility: an international consensus. J Am Med Dir Assoc. 2011;12(6):403-9.

40. Trejo-Avila M, Bozada-Gutiérrez K, Valenzuela-Salazar C, Herrera-Esquivel J, Moreno-Portillo M. Sarcopenia predicts worse postoperative outcomes and decreased survival rates in patients with colorectal cancer: a systematic review and meta-analysis. Int J Colorectal Dis. 2021;36(6):1077-96.

41. Mitchell WK, Williams J, Atherton P, Larvin M, Lund J, Narici M. Sarcopenia, dynapenia, and the impact of advancing age on human skeletal muscle size and strength; a quantitative review. Front Physiol. 2012;3:260.

42. Ibrahim K, Howson FFA, Culliford DJ, Sayer AA, Roberts HC. The feasibility of assessing frailty and sarcopenia in hospitalised older people: a comparison of commonly used tools. BMC Geriatrics. 2019;19(1):42.

43. Schaap LA, van Schoor NM, Lips P, Visser M. Associations of Sarcopenia Definitions, and Their Components, With the Incidence of Recurrent Falling and Fractures: The Longitudinal Aging Study Amsterdam. J Gerontol A Biol Sci Med Sci. 2018;73(9):1199-204.

44. Mijnarends DM, Meijers JM, Halfens RJ, ter Borg S, Luiking YC, Verlaan S, et al. Validity and reliability of tools to measure muscle mass, strength, and physical performance in community-dwelling older people: a systematic review. J Am Med Dir Assoc. 2013;14(3):170-8.

45. Reider L, Owen EC, Dreyer HC, Fitton LS, Willey MC, and M. Loss of Muscle Mass and Strength After Hip Fracture: an Intervention Target for Nutrition Supplementation. Current Osteoporosis Reports. 2023;21(6):710-8.

46. NIHR. Improving inclusion of under-served groups in clinical research: Guidance from the NIHR-INCLUDE project. UK:NIHR2020 [Available from: www.nihr.ac.uk/documents/improving-inclusion-of-under-served-groups-in-clinical-research-guidance-from-include-project/25435

47. Centre NCG. The Management of Hip Fracture in Adults. London: National Clinical Guideline Centre; 2011.

48. Cawthon PM, Peters KE, Cummings SR, Orwoll ES, Hoffman AR, Ensrud KE, et al. Association Between Muscle Mass Determined by D3-Creatine Dilution and Incident Fractures in a Prospective Cohort Study of Older Men. Journal of Bone and Mineral Research. 2022;37(7):1213-20.

49. Pagano AP, Montenegro J, Oliveira CLP, Desai N, Gonzalez MC, Cawthon PM, et al. Estimating Muscle Mass Using D3-Creatine Dilution: A Narrative Review of Clinical Implications and Comparison With Other Methods. The Journals of Gerontology: Series A. 2023;79(4).

50. Hedström M, Ljungqvist O, Cederholm T. Metabolism and catabolism in hip fracture patients: nutritional and anabolic intervention--a review. Acta Orthop. 2006;77(5):741-7.

51. Sanchez-Torralvo FJ, Perez-del-Rio V, Garcia-Olivares M, Porras N, Abuin-Fernandez J, Bravo-Bardaji MF, et al. Global Subjective Assessment and Mini Nutritional Assessment Short Form Better Predict Mortality Than GLIM Malnutrition Criteria in Elderly Patients with Hip Fracture. Nutrients. 2023;15(8):1828.

52. Bachrach-Lindstrom M, Unosson M, Ek AC, Arnqvist HJ. Assessment of nutritional status using biochemical and anthropometric variables in a nutritional intervention study of women with hip fracture. Clinical Nutrition. 2001;20(3):217-23.

53. Cervera-Diaz MDC, Lopez-Gomez JJ, Garcia-Virto V, Aguado-Hernandez HJ, De Luis-Roman DA. Prevalence of Sarcopenia in patients older than 75 years admitted for hip fracture. Endocrinologia, Diabetes y Nutricion. 2023;70(6):396-407.

54. Cha YH, Song SY, Park KS, Yoo JI. Relationship between pressure ulcer risk and sarcopenia in patients with hip fractures. Journal of Wound Care. 2022;31(6):532-6.

55. Cheng SH, Kuo YJ, Lin JCF, Chang WC, Wu CC, Chu YL, et al. Fat distribution may predict intra- or extra-capsular hip fracture in geriatric patients after falling. Injury. 2020;51(2):414-9.

56. Di Monaco M, Castiglioni C, Bardesono F, Milano E, Massazza G. Sarcopenic obesity and function in women with subacute hip fracture: a short-term prospective study. European journal of physical and rehabilitation medicine. 2021;24.

57. Di Monaco M, Castiglioni C, Vallero F, Di Monaco R, Tappero R. Prevalence of sarcopenia following a fracture of the hip is higher in men than in women: A cross-sectional study of 591 patients. Bone. 2012;50:S192.

58. Di Monaco M, Vallero F, Di Monaco R, Tappero R, Cavanna A. Fat mass and skeletal muscle mass in hip-fracture women: a cross-sectional study. Maturitas. 2007;56(4):404-10.

59. Diaz de Bustamante M, Alarcon T, Menendez-Colino R, Ramirez-Martin R, Otero A, Gonzalez-Montalvo JI. Prevalence of malnutrition in a cohort of 509 patients with acute hip fracture: the importance of a comprehensive assessment. European Journal of Clinical Nutrition. 2018;72(1):77-81.

60. González-Montalvo JI, Alarcón T, Gotor P, Queipo R, Velasco R, Hoyos R, et al. Prevalence of sarcopenia in acute hip fracture patients and its influence on short-term clinical outcome. Geriatr Gerontol Int. 2016;16(9):1021-7.

61. Groenendijk I, Kramer CS, den Boeft LM, Hobbelen HSM, van der Putten GJ, de Groot L. Hip Fracture Patients in Geriatric Rehabilitation Show Poor Nutritional Status, Dietary Intake and Muscle Health. Nutrients. 2020;12(9):20.

62. Hicks GE, Shardell MD, Miller RR, Eastlack M, Orwig DL, Goodpaster BH, et al. Trunk Muscle Composition 2 Months After Hip Fracture: Findings From the Baltimore Hip Studies. Archives of Physical Medicine & Rehabilitation. 2019;100(9):1663-71.

63. Hida T, Ishiguro N, Shimokata H, Sakai Y, Matsui Y, Takemura M, et al. High prevalence of sarcopenia and reduced leg muscle mass in Japanese patients immediately after a hip fracture. Geriatr Gerontol Int. 2013;13(2):413-20.

64. Hoekstra JC, Goosen JH, de Wolf GS, Verheyen CC. Effectiveness of multidisciplinary nutritional care on nutritional intake, nutritional status and quality of life in patients with hip fractures: a controlled prospective cohort study. Clinical Nutrition. 2011;30(4):455-61.

65. Jung SY, Kim HJ, Oh KT. Comparative Analysis of Preoperative and Postoperative Muscle Mass around Hip Joint by Computed Tomography in Patients with Hip Fracture. Hip & pelvis. 2022;34(1):10-7.

66. Lau EM, Woo J, Leung PC, Swaminthan R. Low bone mineral density, grip strength and skinfold thickness are important risk factors for hip fracture in Hong Kong Chinese. Osteoporosis International. 1993;3(2):66-70.

67. Malafarina V, Uriz-Otano F, Malafarina C, Martinez JA, Zulet MA. Effectiveness of nutritional supplementation on sarcopenia and recovery in hip fracture patients. A multi-centre randomized trial. Maturitas. 2017;101:42-50.

68. Miller RR, Eastlack M, Hicks GE, Alley DE, Shardell MD, Orwig DL, et al. Asymmetry in CT Scan Measures of Thigh Muscle 2 Months After Hip Fracture: The Baltimore Hip Studies. Journals of Gerontology Series A-Biological Sciences & Medical Sciences. 2015;70(10):1276-80.

69. Park JW, Kim HS, Lee YK, Yoo JI, Choi Y, Ha YC, et al. Sarcopenia: an unsolved problem after hip fracture. J Bone Miner Metab. 2022;40(4):688-95.

70. Yoo JI, Byun H, Kim HS, Jang YJ, Lee CH. Evaluating postoperative muscle strength using surface electromyography in Hip fracture patient. Journal of Bone Metabolism. 2020;27(2):125-32.

70. McGuinness LA, Higgins JPT. Risk-of-bias VISualization (robvis): An R package and Shiny web app for visualizing risk-of-bias assessments. Research Synthesis Methods. 2021;12(1):55-61.
